# Home treatment of COPD exacerbation selected by DECAF score: a non-inferiority, randomised controlled trial and economic evaluation

**DOI:** 10.1136/thoraxjnl-2017-211197

**Published:** 2018-04-21

**Authors:** Carlos Echevarria, Joanne Gray, Tom Hartley, John Steer, Jonathan Miller, A John Simpson, G John Gibson, Stephen C Bourke

**Affiliations:** 1 Respiratory Department, Northumbria Healthcare NHS Foundation Trust, North Shields, UK; 2 ICM, Newcastle University, Newcastle Upon Tyne, UK; 3 Nursing, Midwifery and Health Department, Northumbria University, Newcastle Upon Tyne, UK

**Keywords:** copd exacerbations

## Abstract

**Background:**

Previous models of Hospital at Home (HAH) for COPD exacerbation (ECOPD) were limited by the lack of a reliable prognostic score to guide patient selection. Approximately 50% of hospitalised patients have a low mortality risk by DECAF, thus are potentially suitable.

**Methods:**

In a non-inferiority randomised controlled trial, 118 patients admitted with a low-risk ECOPD (DECAF 0 or 1) were recruited to HAH or usual care (UC). The primary outcome was health and social costs at 90 days.

**Results:**

Mean 90-day costs were £1016 lower in HAH, but the one-sided 95% CI crossed the non-inferiority limit of £150 (CI −2343 to 312). Savings were primarily due to reduced hospital bed days: HAH=1 (IQR 1–7), UC=5 (IQR 2–12) (P=0.001). Length of stay during the index admission in UC was only 3 days, which was 2 days shorter than expected. Based on quality-adjusted life years, the probability of HAH being cost-effective was 90%. There was one death within 90 days in each arm, readmission rates were similar and 90% of patients preferred HAH for subsequent ECOPD.

**Conclusion:**

HAH selected by low-risk DECAF score was safe, clinically effective, cost-effective, and preferred by most patients. Compared with earlier models, selection is simpler and approximately twice as many patients are eligible. The introduction of DECAF was associated with a fall in UC length of stay without adverse outcome, supporting use of DECAF to direct early discharge.

**Trial registration number:**

Registered prospectively ISRCTN29082260.

**Key messages**

**What is the key question?**

- In patients with an exacerbation of COPD triaged for admission, is Hospital at Home directed by low-risk DECAF score (0 or 1) clinically effective and cost-effective compared with usual inpatient care?

**What is the bottom line?**

- Hospital at Home directed by DECAF is safe, clinically effective, cost-effective, and preferred by 90% of patients. This model simplifies selection for Hospital at Home, while approximately doubling the proportion of patients considered eligible compared with previous studies.

**Why read on?**

- The potential clinical and financial benefits of widespread implementation of Hospital at Home directed by DECAF are large, especially given that exacerbation of COPD is the second most common reason for hospital admission.

## Introduction

Hospital at Home (HAH) treats patients in their home for a condition that would otherwise require hospital admission.^1^ The British Thoracic Society,^2^ the National Institute for Health and Care Excellence (NICE)^3^ and the joint European Respiratory Society/American Thoracic Society (ERS/ATS) guidelines^4^ endorse HAH services for patients with COPD exacerbation (ECOPD) and recommend that selection for such services is based on low acute mortality risk. Previous randomised controlled trials (RCTs) of domiciliary care for patients with ECOPD had extensive and inconsistent inclusion and exclusion criteria, partly due to the previous lack of a reliable prognostic score to direct selection of low-risk patients.^5^ The pressing need for prospective research to define optimal criteria for patient selection for HAH has been highlighted.^4^

The DECAF score is a robust predictor of inpatient mortality in patients admitted with ECOPD.^6 7^ It has shown consistent, strong performance in 2645 patients across three cohorts with an area under the receiver operator curve of 0.82–0.86. Of importance, it is simple to score at the bedside using indices routinely available on admission (table 1). The 2014 UK COPD audit report recommends routine documentation of DECAF indices on admission.^8^

**Table 1:** DECAF score

DECAF score		Circle
D*	eMRC**D** 5a (too breathless to leave the house unassisted but independent in washing and/or dressing)	1
	eMRC**D** 5b (t oo breathless to leave the house unassisted and requires help with washing and dressing)	2
E	**E**osinopaenia (eosinophils <0.05×10^9^/L)	1
C	CXR **C**onsolidation	1
A†	Moderate or severe **A**cidaemia (pH<7.3)	1
F	Atrial **F**ibrillation (including history of paroxysmal AF)	1
Total:		

*Breathlessness assessed on a good day within the last 3 months, not breathlessness during an exacerbation/on admission.

†If a blood gas has not been performed, provided oxygen saturation breathing room air is greater than 92%, acidaemia can be assumed not to score. Please refer to the DECAF validation study for detailed instructions on scoring.^7^

AF, atrial fibrillation; CXR, chest radiograph; eMRCD, extended Medical Research Council Dyspnoea score.

Approximately 50% of hospitalised patients have a DECAF score of 0 or 1, which is associated with a low in-hospital mortality risk (1%–1.4%). Selection for HAH by DECAF offers the potential to more than double the proportion of eligible patients compared with earlier models,^5^ while simplifying the selection process. As ECOPD is one of the most common reasons for hospital admission, this represents a large absolute number of patients that could be treated with HAH, but the effect on cost and outcome is unknown.

Accurate prediction of outcome may direct treatment choices and improve outcomes^9^; however, clinical judgement alone is suboptimal.^10^ Before prognostic scores are adopted in routine practice, clinical impact studies assessing outcomes and cost-effectiveness are recommended, although these are seldom performed.^9^ We have undertaken an RCT with an economic evaluation (cost-effectiveness analysis) comparing HAH with usual care (UC) in patients admitted with a low-risk ECOPD selected by DECAF score. The trial examined whether, within a non-inferiority limit of £150, the total health and social care costs up to 90 days associated with HAH are the same or less than those from UC. Clinical outcomes included length of hospital stay (LOHS), readmission rates, mortality and health-related quality of life.

## Methods

### Study design and patients

In a non-inferiority RCT, eligible patients with a low-risk (DECAF 0–1) ECOPD^11^ admitted to one of three hospitals within one Trust underwent 1:1 allocation to HAH or UC and were followed for 90 days from presentation. In the UK healthcare system, a National Health Service Trust is an organisation that serves a geographical region, in this instance a socioeconomically diverse urban and rural population, with the largest geographical footprint in England. The COPD population has high rates of social deprivation and comorbidity.^7 8^ Ninety days was chosen for the primary outcome because this is the key risk period for readmission.^12^

Eligibility criteria included low mortality risk (DECAF 0–1), age 35 years or older, 10 or more smoking pack-years, and pre-existing or admission obstructive spirometry.^11^ Inpatient spirometry was only performed in individuals with a pre-existing COPD diagnosis where confirmatory spirometry was unavailable (eg, inaccessible general practitioner (GP) records on weekends) or in those with a high pretest probability of a new diagnosis of COPD. Patients were excluded if they had an illness (other than COPD) likely to limit survival to less than 1 year, were on long-term ventilation, had a coexistent secondary diagnosis necessitating admission, were assessed more than one overnight stay after admission or could not provide written informed consent. Patients were not eligible to enter the trial from the emergency department to ensure only admitted patients were included.

All patients who met the entry criteria were offered participation, including DECAF 1 patients with coexistent pneumonia or acidaemia. All patients were analysed in their original allocated group, even if the consultant decided that an HAH patient should stay in the hospital. Baseline data were collected prior to treatment allocation. In the HAH arm, patients readmitted during follow-up with a low-risk ECOPD were offered HAH while all other readmissions were managed according to UC.

### Randomisation and masking

Allocation to HAH or UC was based on 1:1 randomisation, performed by minimisation^13^ (table 2) undertaken by an external, independent agency (sealedenvelope.com). Individual patients had a 30% chance of allocation purely by random number sequence; the researchers were blind to the method of allocation for individual patients. For the primary cost analysis, the health economist was blinded to group allocation.

**Table 2:** Minimisation indices

ABG (management pathway)	PaCO_2_ ≤6 + pH ≥7.35	PaCO_2_ >6 + pH ≥7.35	pH <7.35
Hospital admissions in the previous year	0	1	2 or more
Prior social care (private or social services)	None	Social care	
eMRCD score	1–4	5a	
Cerebrovascular disease	Yes	No	

ABG, arterial blood gas; eMRCD score, Extended Medical Research Council Dyspnoea score.

### Procedures

#### HAH treatment

HAH treatment replaces all or most of the hospital admission and requires that patients are not sufficiently well for discharge, resulting in a more unwell population than seen in early supported discharge (ESD) services.

In our HAH model, patients were admitted to hospital, identified as low risk by DECAF, and then returned home under the care of the hospital respiratory team, usually within 24 hours of admission. The HAH treatment period ended when the respiratory specialist nurse (RSN) and consultant deemed that the patient was sufficiently well for discharge to the care of the GP, typically after 5 days.

Patients received once or twice daily visits from an RSN, under remote supervision from a respiratory consultant. An emergency contact number allowed patients to contact the team 24 hours a day, 7 days a week. Physiological parameters were monitored daily and blood sampling (including arterial blood gas analysis) taken as required. Oral and intravenous therapies, acute controlled oxygen therapy, physiotherapy, psychology, occupational therapy and formal social care were available at home.

Patients randomised to HAH could return home immediately provided the initial arterial pH was 7.35 or more and PaCO_2_ was 6 kPa or less. Patients with PaCO_2_ greater than 6 kPa without acidaemia could return home after one overnight stay in hospital, provided they were not deteriorating. Patients with acidaemia could return home the day that followed resolution of the acidaemia and, if initiated, once non-invasive ventilation was complete. This ‘ABG management pathway’ was included as one of the minimisation indices.

Return to hospital during HAH was not considered a readmission, but rather an increase in level of care. If return to hospital during HAH were considered a readmission, this could create bias because patients in UC are hospitalised and therefore not exposed to the risk of readmission.

Further details of the HAH service are available in the HAH manual and review sheets in online supplementary files 1, 2 and 3. The manual has been updated following service feedback, but the interventions and procedures are unchanged from those used in the trial.

#### Usual care

This included usual measures to ensure the prompt discharge of patients with ECOPD, such as supported discharge by RSNs. Based on local data from 492 patients scoring DECAF 0 or 1 prior to the trial, we anticipated that the median LOHS would be 5 days. The decision to discharge patients in the UC group was made by the attending clinician.

### Outcomes

The primary outcome was the total cost of health and formal social care over 90 days from presentation, costed from a UK health and social care perspective. The secondary outcomes were survival, readmission rate, total bed days over 90 days and cost-effectiveness, using the EuroQuality of life instrument (EQ-5D-5L) quality-adjusted life year (QALY) measured at baseline, 14 and 90 days,^14^ patient preference for HAH or UC (as a binary question at 14 days), COPD exacerbations, Hospital Anxiety and Depression Scale scores (HADS), and COPD Assessment Tool (CAT) scores.

All costs, unless stated otherwise, were recorded at the patient level by multiplying patient-level resource use by the appropriate unit cost, and the average costs per treatment arm were subsequently estimated. Data collection was the same in both arms, except for resource collection during HAH treatment (‘HAH visits and travel time’ and ‘telephone call costs’). All visiting health and social care staff recorded time spent with the patient and travel time, including interactions outside of usual work hours. This was triangulated with a time and motion study performed by RSNs in a subpopulation of HAH patients.

Patients in both arms maintained a diary of all health and social care visits and attendances, and were phoned every 2 weeks to prompt completion and collect data. These data were cross-referenced with primary, secondary and social care records to provide costs for ‘formal social care’, ‘home visits after discharge’ and ‘A+E and outpatient appointments’. Additional consent was gained for remote monitoring of health and social records if the patient withdrew from the trial, allowing complete data capture.

For primary care, resource use included all medications, GP appointments, and home visits by doctors and allied healthcare professionals.

Secondary care inpatient costs considered specific to DECAF 0–1 patients were costed at the patient level. This included inpatient healthcare reviews, medications, laboratory and diagnostic costs, oxygen use, non-invasive ventilation use and LOHS. All ‘inpatient healthcare reviews’ were recorded, including those by doctors, specialist nurse and physiotherapists; this was costed based on the seniority of the individual and the amount of time spent with the patient. Where unavailable, the time spent with the patient was estimated based on the type of encounter (such as ‘physiotherapy chest clearance’) and the average time taken for similar encounters; all assumptions were the same across both arms, and assumptions regarding the type of encounter were performed blind to group allocation.

The remaining inpatient costs are those that we expected would be similar between patients and/or were not possible to separate out at the patient level, for which an average bed day cost was calculated. The cost of a day on a ward was costed using data from the Trust’s finance department. This included running costs (including catering, laundry, gas and electricity), staff costs (such as support staff), equipment (medical, surgical and non-medical) and associated services (such as phlebotomy). These costs were not patient-specific and were assumed the same regardless of patients’ characteristics. This was performed separately to give a cost for the admissions unit, medical ward and rehabilitation ward.

All outpatient visits and accident and emergency attendances were recorded.

Social care resource use, including formal social care and equipment costs, was obtained from individual social care records.

Unit costs were obtained from a variety of national and local sources and are reported in online supplementary file 4 for the financial year 2015 (£), with key unit costs shown in table 3.

**Table 3:** Key unit costs

Type of unit cost	Source	Cost (£)
A+E attendance	NHS reference costs 2015	90.2–377.9
Outpatient clinics	NHS reference costs 2015	39.7–215.4
Respiratory clinic	NHS reference costs 2015	165.9
Bed days, admissions unit	Healthcare Trust	294.9 per day
Bed days, medical ward	Healthcare Trust	246.2 per day
Bed days, rehabilitation ward	Healthcare Trust	168.8 per day
Doctor, consultant time	PSSRU unit costs of health+social care 2015	153 per hour
Doctor, registrar time	PSSRU unit costs of health+social care 2015	68.4–75 per hour
Doctor, F1–ST2	PSSRU unit costs of health+social care 2015	42.5–67 per hour
Respiratory specialist nurse	PSSRU unit costs of health+social care 2015	46.2–68.6 per hour
Physiotherapy	PSSRU unit costs of health+social care 2015	39.2–49.5 per hour

A+E, accident and emergency; NHS, National Health Service; PSSRU, personal social services research unit.

### Statistical analysis

The primary outcome was the mean difference between HAH and UC in total health and social care costs over 90 days. HAH was deemed non-inferior to UC if the upper limit of the one-sided 95% CI for the primary outcome was less than the non-inferiority limit. CIs were calculated with 1000 bootstrap replications. For the breakdown in costs (table 7), two-sided 95% CIs were calculated.

The non-inferiority limit and the power calculation were based on the best available data, which were limited to health costs for the index admission. Based on tariff costs received by the Trust for 373 patients admitted with DECAF 0–1 ECOPD, the estimated SD of costs was £1143, and HAH costs were estimated as £470 less expensive per patient compared with UC. One hundred and eighteen patients were required to be 90% sure that the upper limit of a one-sided 95% CI would be below the non-inferiority limit of £150, if the true difference in costs were 0.^15^ This threshold was discussed with hospital managers, who confirmed that if HAH was £150 more expensive than UC this would not prevent them from financially supporting the implementation of HAH services.

The outcome measure used in the economic analysis was the QALY. Health-related quality of life was assessed using the EQ-5D-5L questionnaire, which is valid and responsive in COPD,^16^ and a standard algorithm was used to obtain utility scores.^17^ The QALY was obtained by linear regression estimation, controlling for intervention groups and baseline utility using the area under the curve approach (individual QALYs were calculated by taking the mean value between measurements and multiplying this with time).^18^ The cost-effectiveness plane and a cost-effectiveness acceptability curve were derived from the joint distribution of incremental costs and incremental QALYs using non-parametric bootstrapping of the observed data.

Bed days were compared using Mann-Whitney U test with a two-sided P value of <0.05 regarded as significant. Primary analyses were performed with complete case analysis. A sensitivity analysis was performed using multiple imputation, with missing data assumed to be missing at random, to create five data sets^19^ using the Markov chain Monte Carlo method. All baseline patient characteristics and outcomes were included in the imputation model. Data were analysed using IBM SPSS V.22 statistics and Stata V.14. Patients allocated to HAH who received UC treatment were analysed in their original allocation group as per the intention-to-treat principle. In a prespecified safety analysis, deaths and readmissions were reported per protocol.

The funders had no role in data collection, analysis or in writing of the report. During the review process, we agreed to make our prespecified cost outcome the primary measure, replacing total bed days over 90 days.

## Results

**Figure 1:**
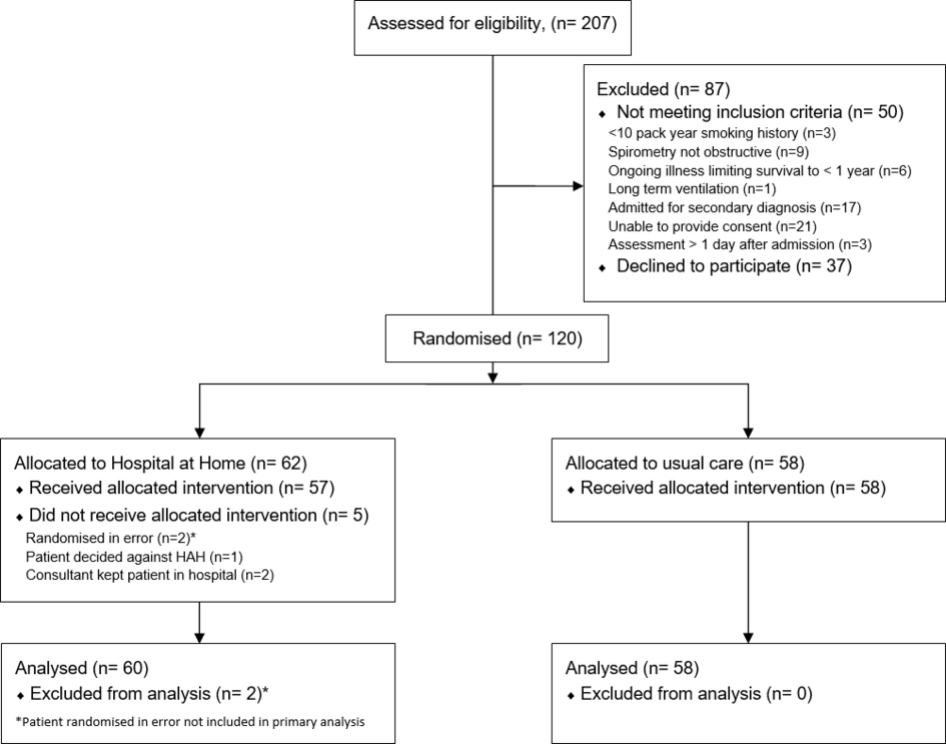
CONSORT diagram. CONSORT, Consolidated Standards of Reporting Trials; HAH, Hospital at Home.

Emergency hospital admissions from June 2014 to January 2016 were reviewed to ensure all patients with ECOPD were identified. Of note, 64 patients with a DECAF 0–1 ECOPD were planned for same-day discharge before eligibility assessment and were not included because HAH is not indicated for those who are sufficiently well for discharge. Of 207 DECAF 0 or 1 ECOPD assessed for eligibility, 120 were randomised. Two patients who did not meet the eligibility criteria were randomised in error and were not included in the primary analysis. In both instances, this was recognised and the patients were removed within 30 min of randomisation. Three patients were randomised to HAH, but were intentionally treated by UC, and were analysed in their original allocation as per the intention-to-treat principle (see figure 1). Groups were well matched with respect to minimisation indices (table 4).

**Table 4:** Baseline characteristics of patients

	HAH, n=60	UC, n=58
DECAF indices		
DECAF score 1, n (%)	43 (71.7)	31 (53.4)
eMRCD dyspnoea score 5a, n (%) repeat below	12 (20)	9 (15.5)
Eosinopaenia, %	15 (25)	8 (13.8)
CXR consolidation, %	15 (25)	9 (15.5)
Acidaemia (pH <7.30), %	1 (1.7)	0
Atrial fibrillation, %	0	0
Minimisation indices		
ABG management, pH <7.35 / PCO_2_ >6 pH ≥7.35, %	7 (11.7) / 40 (66.7)	8 (13.8) / 38 (65.5)
Hospital admissions in the previous year 1 / 2, %	12 (20) / 21 (35)	12 (20.7) / 19 (32.8)
Prior social care, %	3 (5)	1 (1.7)
Cerebrovascular disease, %	9 (15)	9 (15.5)
Sociodemographics		
Age, years*	71.0 (9.6)	68.7 (10.5)
Female, %	32 (53.3)	30 (51.7)
Smoking pack-years, n†	45 (35–50)	44 (30–60)
Current smoking, %	27 (45)	25 (43.1)
Reporting no qualifications on leaving school, %	46 (76.7)	41 (70.7)
Most frequently reported family income per year, £†	5200–10 399	10 400–15 599
Markers of disease severity		
FEV_1_% predicted*	45.5 (18.4)	42.1 (16.3)
LTOT prior to admission, %	7 (11.7)	2 (3.4)
Cor pulmonale, %	11 (18.3)	5 (8.6)
Comorbidity		
IHD, %	14 (23.3)	12 (20.7)
Diabetes, %	8 (13.3)	5 (8.6)
LVD, %	1 (1.7)	3 (5.2)
Anxiety, %	9 (15.0)	3 (5.2)
Depression, %	12 (20.0)	9 (15.5)
Admission clinical data		
Respiratory rate, per minute*	25 (4.5)	26 (5.1)
Pulse rate, per minute*	103.9 (19.6)	104.9 (15.4)
sBP, mm Hg*	140.8 (21.1)	145.1 (24.3
dBP, mm Hg*	77.3 (12.2)	80.9 (14.5)
Temperature, °C†	36.6 (36.2–37.3)	36.5 (36.1–37.1)
Oxygen saturation†	92 (89–94)	92 (88.5–95)
Discoloured sputum, %	43 (71.7)	33 (56.9)
Arterial blood gas values		
pH†	7.42 (7.39–7.45)	7.42 (7.38–7.44)
PaO_2_, kPa†	7.6 (7.2–9.3)	7.9 (7.2–10.2)
PaCO_2_, kPa†	5.5 (5–6.25)	5.3 (4.8–6.6)
HCO_3_, mmol/L*	27.1 (4.3)	27.3 (4.7)
pH <7.35, %	7 (11.7)	8 (13.8)
Baseline outcome measures		
Utility score (EQ-5D-5L), n*	0.517 (0.268)	0.501 (0.243)
Hospital Anxiety and Depression Scale score A / D, n†	6 (4–10.25) / 7 (4–9)	7 (4–10) / 5 (2–8.25)
COPD Assessment Tool, n†	28.5 (21.75–33)	27 (23–32.25)
Treatment		
ECOPD treatment prior to admissions, %	32 (53.3)	26 (44.8)

*Mean (SD).

†Median (IQR).

A/D, anxiety/depression; ABG, arterial blood gas; CXR, chest radiograph; dBP, diastolic blood pressure; ECOPD, COPD exacerbation; eMRCD, extended Medical Research Council Dyspnoea score; EQ-5D-5L, EuroQuality of life; HAH, Hospital at Home; HCO_3_, bicarbonate; IHD, ischaemic heart disease; LTOT, long-term oxygen therapy; LVD, left ventricular dysfunction; sBP, systolic blood pressure; UC, usual care.

### Clinical outcomes

There were no deaths in the acute period (within 14 days) in either arm. Within 90 days, there was one death in each arm. There was a statistically significant reduction in bed days over 90 days in those treated with HAH (HAH=1, IQR 1–7 compared with UC=5, IQR 2–12; P=0.001). Readmission rates were similar in both arms, with 22 (36.7%) in HAH and 23 (39.7%) in UC (table 5).

**Table 5:** Mortality, length of stay, readmission, appointments and social care, and treatment preference outcome

	HAH, n=60	UC, n=58
Death at 14 days, n (%)	0 (0)	0 (0)
Death at 90 days, n (%)	1 (1.7)	1 (1.7)
Length of hospital stay at 90 days, median (IQR)	1 (1–7)	5 (2–12)*
Length of hospital stay at 90 days, mean (SD)	6.1 (9.7)	10.3 (15.8)
Length of hospital stay (index admission), median (IQR)	1 (1–1)	3 (2–4.25)
Length of hospital stay (index admission), mean (SD)	1.2 (2.1)	4.1 (4.6)
Length of stay within HAH, median (IQR)	4 (2–5)	NA
Patients with one or more hospital readmissions, n (%)	22 (36.7)	23 (39.7)
Patients with one or more A+E attendances post discharge, n (%)	29 (48.3)	26 (44.8)
Patients with one or more GP attendances post discharge, n (%)	26 (43.3)	30 (51.7)
Patients with one or more secondary care appointments, n (%)	48 (80.0)	41 (70.7)
Patients with a social care package post discharge, n (%)	7 (11.7)	5 (8.6)
Stated preference for HAH care day 14, n (%)	54 (90.0)	51 (87.9)

*P=0.001 using Mann-Whitney. For bed days over 90 days, based on length of stay from 373 patients, 116 patients were needed to detect a difference of 4.7 days with 90% power assuming a type 1 error rate of 5% in a superiority analysis.

A+E, accident and emergency; GP, general practitioner; HAH, Hospital at Home; NA, not applicable; UC, usual care.

At 14 days, 90% of patients across both arms stated they would prefer HAH treatment during future exacerbations of similar severity (HAH=54 of 60; UC=51 of 57). In the prespecified, per-protocol safety analysis, deaths were unchanged (one in each arm at 90 days), and there were 21 of 57 (36.8%) readmissions in HAH and 23 in 58 (39.7%) in UC. Table 6 shows the change in quality of life scores from baseline at 14 days and 90 days as the unit change and as the per cent of patients who improved by a minimally clinically important difference (MCID). Further data on utility scores are available in online supplementary table e10.

**Table 6:** Changes in quality of life and HADS scores from baseline

	HAH			UC		
	Unit change*	% MCID†	Missing	Unit change	% MCID	Missing
HADS-A 14-day (IQR)	−1.0 (−3 to 1.75)	48.3	0	0.5 (−3 to 2)	33.9	2
HADS-A 90-day (IQR)	0 (−2 to 3)	33.3	6	0 (−3 to 2)	38.2	3
HADS-D 14-day (IQR)	−1.0 (−3 to 1)	38.3	0	0 (−2 to 3)	26.8	2
HADS-D 90-day (IQR)	−0.5 (−3 to 1.25)	37.0	6	0 (−2 to 3)	27.3	3
CAT 14-day (IQR)	−4.0 (−9.5 to 0)	61.7	0	−3.0 (−7 to 1)	57.9	1
CAT 90-day (IQR)	−3.0 (−8 to 1)	51.9	6	−1.0 (−6 to 1)	36.4	3
Utility 14-day (EQ-5D-5L) (SD)	0.091 (0.249)	56.7	0	0.055 (0.316)	49.1	1
Utility 90-day (EQ-5D-5L) (SD)	0.003 (0.287)	43.9	3	0.007 (0.338)	41.1	2

*Values are median, except utility which is mean. Unit change is the difference in absolute values between follow-up and baseline. Improvements in health status are negative for HADS and CAT, and positive for utility scores.

†The percentage of patients who improved by an MCID, which is 1.5 for HADS-A and HADS-D,^21^ 2 for CAT and 0.051 for the EQ-5D-5L.^20^

CAT, COPD Assessment Tool; EQ-5d-5L, Euro Quality of life instrument; HADS, Hospital Anxiety and Depression Scale; HAH, Hospital at Home; MCID, minimally clinically important difference; UC, usual care.

For HADS and CAT, negative values represent improvements in health from baseline, while for utility scores, positive values represent improvements from baseline. The improvements in health status in HAH compared with UC were clinically meaningful for HADS-anxiety score at 14 days and CAT at 90 days, but this could be a chance finding.^20 21^ On multiple imputation the difference in the benefit of CAT at 90 days was 1.5, but the utility score at 14 days was 0.51, which is above the MCID (online supplementary table e11).

### Cost and cost-effectiveness analysis

**Figure 2:**
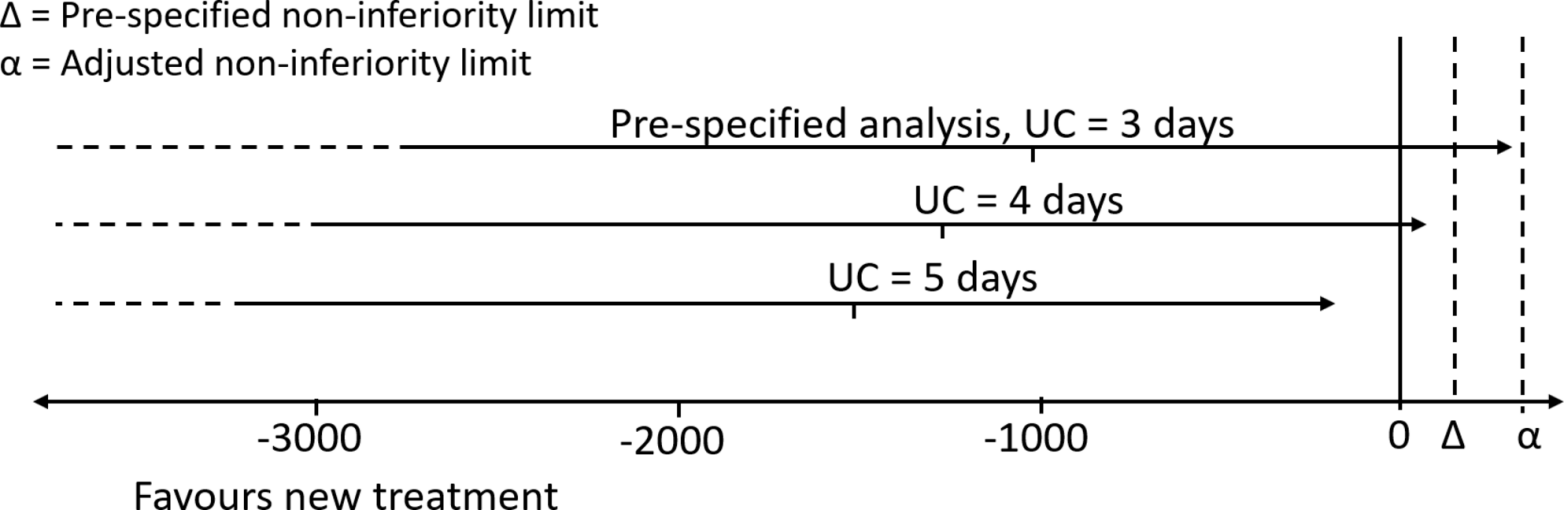
Length of stay and cost difference (£) between HAH and UC. One-sided CIs for the mean difference in 90-day health and social care costs between UC and HAH are shown for the trial population (UC=3 days) and the sensitivity analysis adjusting for a longer hospital stay in UC (UC=4 days and UC=5 days); Δ=£150, α=£340. HAH, Hospital at Home; UC, usual care.

The mean health and formal social care cost over 90 days was £1016 lower in HAH than in UC. However, there was wide variation in costs and the one-sided 95% CI crossed both the no effect limit (0) and the prespecified non-inferiority limit of £150 (figure 2, ‘UC=3 days’: CI −2343 to 312). The cost difference and distribution were substantially greater than anticipated, and so a post-hoc analysis was performed with an adjusted non-inferiority limit of £340,^15^ which was achieved (see figure 2 and the Discussion section).

During the index admission, the median LOHS in UC was 3 days, which was 2 days less than expected^7^ and greater than seen in most UK hospitals for unselected ECOPD.^8^ We performed a prespecified sensitivity analysis to assess the effect of LOHS in UC during the index admission on health and formal social care costs. One additional bed day without medical staffing costs would increase the mean cost difference to −£1262 with a one sided 95th percentile of £66, achieving the prespecified non-inferiority limit of £150. Two bed days would have been −£1508 with a one-sided 95th percentile of −£180.

**Figure 3:**
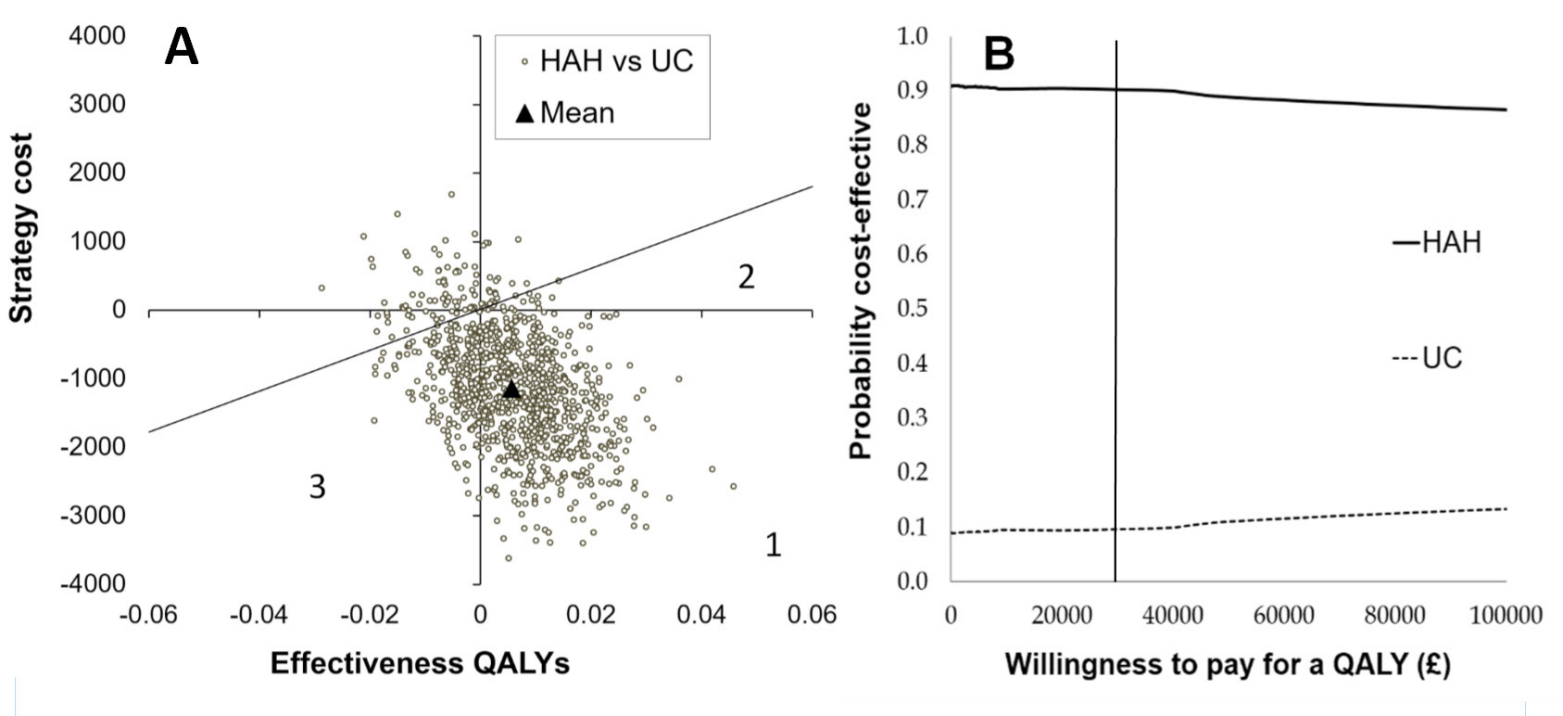
Cost-effectiveness plane (A) and cost-effectiveness acceptability curve (B). The cost-effectiveness plane for HAH and UC, with the diagonal line representing the NICE cut-off at £30 000 per QALY. Area 1=HAH cheaper and more effective; area 2=HAH more effective and more expensive but less than the NICE cut-off; and area 3=UC is more effective but more expensive and exceeds the NICE cut-off. (B) The probability of cost-effectiveness is shown over a range of willingness to pay for a QALY, to inform decisions to accept or reject new technologies. There is a 90% probability HAH will be cost-effective at the NICE threshold (vertical line). HAH, Hospital at Home; NICE, National Institute for Health and Care Excellence; QALY, quality-adjusted life year; UC, usual care.

The difference in cost was primarily related to inpatient and formal social services costs (table 7). The costs of the index admission alone are shown in online supplementary table e12. Total QALY scores were non-significantly higher in HAH compared with UC. The mean total QALYs (SD) adjusted for baseline utility were 0.138 (0.052) for HAH and 0.133 (0.052) for UC, giving a small difference of 0.005 (95% CI −0.14 to 0.25). Unadjusted and Multiple Imputation (MI) analyses of QALYs are shown in online supplementary table e10. The probability of HAH being cost-effective compared with UC was 90% at the NICE threshold of £30 000 per QALY. This is the proportion of dots beneath the diagonal line in figure 3A, and is represented by the vertical line in figure 3B. HAH was both cheaper and more effective for most patients treated (74% probability). Similar results were seen using multiple imputation (online supplementary figure E1). Of note, the Cost Effectiveness plane shows high uncertainty around the incremental cost difference, although little uncertainty around the incremental effectiveness estimates.

**Table 7:** Health and formal social care average costs at 90 days

Overall costs	HAH, £ (SD)	UC, £ (SD)	Bootstrapped mean difference (£)	Bootstrapped 95% CI* of cost difference
Health and formal social care	3857.8 (3199.6)	4873.5 (5631.1)	−1015.7	−2735.5 to 644.8
Healthcare	3819.2 (3135.0)	4755.8 (5525.4)	−936.6	−2645.4 to 709.9
Oxygen therapy	38.4 (68.4)	18.3 (53.7)	20.1	−1.73 to 42.0
Medication	422.5 (275.2)	458.9 (331.4)	−36.4	−150.1 to 75.7
Hospital costs				
Bed stay	1540.8 (2000.7)	2775.2 (4129.6)	−1234.4	−2524.8 to −82.0
Inpatient healthcare review	417.7 (399.1)	514.3 (650.7)	−96.7	−288.4 to 96.4
Laboratory and diagnostic tests	375.1 (383.8)	358.7 (422.4)	16.4	−128.1 to 169.1
NIV costs	44.4 (261.0)	158.2 (436.2)	−113.8	−255.4 to 8.12
HAH costs				
HAH visits and travel time†	383.9 (276.0)	0.0 (0.0)	383.9	319.2 to 455.3
Telephone calls costs	5.8 (14.2)	5.4 (10.8)	0.5	−3.57 to 5.33
Community costs				
Formal social care	38.6 (173.1)	117.7 (711.0)	−79.0	−299.2 to 55.2
Home visits after discharge	43.7 (87.7)	39.2 (55.7)	4.5	−19.2 to 31.8
A+E and outpatient appointments	546.8 (347.5)	427.6 (394.9)	119.2	−22.6 to 243.0

*The 95% CI in the table is two-sided (0.025 to 0.975), calculated with the bootstrap approach. For health and formal social care (the primary outcome), the one-sided 95% CI (0.95) was £312.

†55% of time on HAH visits was spent with the patient (45% on travel time).

A+E, accident and emergency; HAH, Hospital at Home; NIV, non-invasive ventilation; UC, usual care.

### HAH and inpatient interactions

Of the 60 patients allocated to HAH, 53 (88.3%) had a 0 or 1 day stay. Most patients incurring an overnight stay were admitted in the afternoon or evening. The period of HAH lasted a median of 4 (IQR 2–5) days per episode. Including travel time, healthcare professionals spent a median of 7.2 hours (IQR 4.7–10.8) on home visits per HAH spell (median RSN visits=7.1 hours, IQR 4.4–10.1). There were 342 visits for 57 episodes: RSN=327, physiotherapy=13, psychology=1 and respiratory support worker=1. During HAH, two patients returned to hospital for assessment (which included a respiratory consultant review, repeat chest radiograph and blood testing) and returned home the same day. One patient returned to hospital and stayed overnight before returning home to complete their HAH spell.

The number of inpatient interactions with any healthcare worker was 1158 for HAH (1500 including inpatient interactions, or 25 interactions per patient) and 1558 for UC (or 27 interactions per patient). In part, the increased numbers of inpatient interactions for UC were due to reviews by doctors and physiotherapists (see online supplementary table e13).

### Patients declining participation

As part of an audit of practice, the baseline characteristics of patients who declined to participate in the HAH study were reviewed. Patients who declined enrolment were not more unwell than study participants based on comorbidity and measures of disease severity (online supplementary table e14).

## Discussion

In an economic evaluation, HAH selected by DECAF was more cost-effective than UC, primarily driven by a fivefold reduction in median hospital bed days over 90 days, with a small non-significant difference in QALYs favouring HAH. The percentage of patients improving by the MCID was numerically higher in HAH compared with UC for seven of eight outcomes measuring health status.

The potential cost savings are substantial as ECOPD is one of the most common reasons for hospital admission and up to 50% of patients are potentially eligible (DECAF 0–1). In both arms, there were no deaths within the acute period, and readmission rates over 90 days were comparable in intention-to-treat and per-protocol analyses. Crucially, 90% of patients across both arms stated they would prefer HAH to UC for future exacerbations of similar severity. The DECAF score allows low-risk patients to be identified quickly and safely using indices routinely captured on admission, facilitating replication of our model of HAH. This meets the major research need identified by the ERS/ATS to better define patient selection criteria for HAH.^4^ Of importance, use of DECAF was associated with reduction in LOHS within UC of at least 2 days, without adverse outcome. This supports use of a low-risk DECAF score to select patients for early discharge, which may be implemented in advance of establishing a full HAH clinical service.

This study has several strengths. We assessed the impact of using the DECAF score to direct HAH treatment, replicating how we anticipate the tool will be used in clinical practice. Such implementation studies are extremely rare despite being strongly recommended.^9^ We performed a detailed and extensive cost analysis, recording all important aspects of health and social care with low rates of missing data. We included several important measures of health status which the ERS/ATS reported is lacking in previous studies,^4^ and methods of patient allocation and handling of missing data were robust. Patients were randomised by minimisation, which ensures excellent balance for selected prognostic indices.^13 22^ The likelihood of allocation to an intervention is influenced by the current distribution of subjects and weighted minimisation indices. To avoid potential selection bias, 30% of allocations were by simple randomisation and the researchers were blinded to the allocation process, performed by an independent agency. The HAH service included all members of the usual multidisciplinary team and important aspects of care such as smoking cessation, inhaler training, breathing exercises and the offer of early pulmonary rehabilitation.

One of the key limitations of the study was the choice of £150 as the non-inferiority limit, which meant that HAH did not meet the chosen non-inferiority limit. First, this occurred because the data were only available for a single admission, and not for the primary outcome of total health and social care cost over 90 days. The actual mean total cost over 90 days in UC was far higher than anticipated at £4874, so a non-inferiority limit of £150 was overly conservative. It is usual in non-inferiority studies to choose a margin that reflects the largest loss that would be acceptable.^23^ In the context of a higher mean difference, a larger non-inferiority limit is appropriate. Non-inferiority limits should be based on statistical reasoning and clinical judgement. In our post-hoc analysis a non-inferiority limit to £340 was selected. Statistically, we chose this value as it is one-third of the cost difference between arms, which is the same ratio as the original non-inferiority limit and estimated cost difference. The acceptability of this non-inferiority limit is confirmed by the fact that this model of HAH has subsequently been commissioned. Second, the cost difference between HAH and UC may have been affected by a reduction in LOHS in UC. The number of patients unsuitable for HAH (because they already had same-day discharge plans) was larger than expected, resulting in a more unwell and costly study population. This should have resulted in an increased median LOHS in UC, but it was 2 days lower than expected. Non-exclusion of more unwell patients with longer LOHS could theoretically account for this. However, this is unlikely as the short stay group (n=64) was larger than the excluded group (n=50), and would have had a bigger impact on the median value. Furthermore, those who declined participation in the study were not more unwell than study participants. The most likely explanation is that the use of the DECAF score and study participation reduced LOHS. Only UC patients expressed disappointment with their allocated arm, knowledge of participation may have influenced clinician behaviour and bed pressures may have exerted additional influence.

Despite a large proportion of patients improving by the MCID (table 6), baseline and 90-day follow-up quality of life scores were similar across the whole population. This apparent discrepancy may be explained by worsening health status in those who were readmitted. In those who suffered an overall deterioration in utility score at 90 days, the proportion with one or more admissions was 2.5-fold higher.

The results of the study require validation in other healthcare systems. The structure of care, including availability of ESD, differed between sites and the DECAF score has previously effectively identified low-risk patients in six different hospitals, with different populations and structures of care.^7^ This supports the generalisability of the results to other UK hospitals. Some hospitals may currently lack the nursing infrastructure to deliver HAH selected by DECAF, but investment is justified as there is a 90% chance of this model being cost-effective at both the NICE and commonly cited US thresholds, with further possible cost savings through reduced LOHS in UC. Training costs of nurses were included in our analysis. Finally, 90-day follow-up was selected because this is the critical time period for readmission,^12^ although a longer time period of up to 1 year may have been preferable to identify a difference in readmission rates between groups.

Meta-analyses of previous studies considered HAH and ESD together. These showed that HAH/ESD report reduced readmission rates and a trend towards a lower mortality with limited evidence for an effect on health-related quality of life.^5 24^ Three studies performed cost analyses showing that HAH/ESD was less expensive.^25–27^ Goossens^28^ and others performed a detailed economic evaluation: at 3 months HAH/ESD was €168 less expensive than UC from a healthcare perspective, but €908 more expensive when societal costs were included. These previous studies are primarily of ESD services rather than HAH, and comparison with our study should be guarded. For example, in the study by Goossens and others, length of stay in the ESD treatment arm was the same as our UC arm.

Previous studies of HAH/ESD had extensive eligibility criteria to identify suitable, low-risk patients and typically excluded those with coexistent pneumonia and acidaemia on blood gas.^25 26 29–33^ Ordinarily, clinicians would be reluctant to allow these patients into HAH/ESD services, but we treated such patients successfully with no difference in mortality between groups. This result is consistent with findings from the DECAF derivation and validation study, which showed that patients with a low-risk DECAF score and pneumonia or acidaemia had a low acute mortality risk.^6 7^

This RCT shows that HAH selected by low-risk DECAF score is safe, clinically effective, preferred by most patients and cost-effective compared with UC in this clinical setting. DECAF has proven a robust tool in the gold standard of derivation, validation and implementation studies, and can be used in clinical practice to select low-risk patients for HAH services. Based on this result, our commissioners and the Trust have agreed to the implementation of a full clinical service.

10.1136/thoraxjnl-2017-211197.supp1 Supplementary file 1

10.1136/thoraxjnl-2017-211197.supp2 Supplementary file 2

10.1136/thoraxjnl-2017-211197.supp3 Supplementary file 3

10.1136/thoraxjnl-2017-211197.supp4 Supplementary file 4

## References

[1] Shepperd S, Doll H, Broad J, et al Hospital at home early discharge. Cochrane Database Syst Rev 2009:CD000356. 19160179 10.1002/14651858.CD000356.pub3 PMC4175532

[2] British Thoracic Society Guideline Development Group. Intermediate care–Hospital-at-Home in chronic obstructive pulmonary disease: British thoracic society guideline. Thorax 2007;62:200–10. 17090570 10.1136/thx.2006.064931 PMC2117156

[3] National Clinical Guideline Centre. Chronic obstructive pulmonary disease: management of chronic obstructive pulmonary disease in adults in primary and secondary care. London: National Clinical Guideline Centre, 2010.

[4] Wedzicha JA, Calverley PMA, Albert RK, et al Prevention of COPD exacerbations: a European Respiratory Society/American Thoracic Society guideline. Eur Respir J 2017;50:1602265 10.1183/13993003.02265-2016 28889106

[5] Echevarria C, Brewin K, Horobin H, et al Early supported discharge/hospital at home for acute exacerbation of chronic obstructive pulmonary disease: a review and meta-analysis. COPD 2016;13:523–33. 10.3109/15412555.2015.1067885 26854816

[6] Steer J, Gibson J, Bourke SC The DECAF Score: predicting hospital mortality in exacerbations of chronic obstructive pulmonary disease. Thorax 2012;67:970–6. 10.1136/thoraxjnl-2012-202103 22895999

[7] Echevarria C, Steer J, Heslop-Marshall K, et al Validation of the DECAF score to predict hospital mortality in acute exacerbations of COPD. Thorax 2016;71:133–40. 10.1136/thoraxjnl-2015-207775 26769015 PMC4752621

[8] Stone RA, Holzhauer-Barrie J, Lowe D, et al COPD: Who cares matters. National Chronic Obstructive Pulmonary Disease (COPD) Audit Programme. Clinical audit of COPD exacerbations admitted to acute units in England and Wales 2014, 2015.

[9] Steyerberg EW, Moons KG, van der Windt DA, et al Prognosis research strategy (PROGRESS) 3: prognostic model research. PLoS Med 2013;10:e1001381 10.1371/journal.pmed.1001381 23393430 PMC3564751

[10] Wildman MJ, Sanderson C, Groves J, et al Predicting mortality for patients with exacerbations of COPD and Asthma in the COPD and Asthma Outcome Study (CAOS). QJM 2009;102:389–99. 10.1093/qjmed/hcp036 19369483

[11] Vestbo J, Hurd SS, Agustí AG, et al Global strategy for the diagnosis, management, and prevention of chronic obstructive pulmonary disease: GOLD executive summary. Am J Respir Crit Care Med 2013;187:347–65. 10.1164/rccm.201204-0596PP 22878278

[12] Echevarria C, Steer J, Heslop-Marshall K, et al The PEARL score predicts 90-day readmission or death after hospitalisation for acute exacerbation of COPD. Thorax 2017;72:686–93. 10.1136/thoraxjnl-2016-209298 28235886 PMC5537524

[13] Altman DG, Bland JM Treatment allocation by minimisation. BMJ 2005;330:843 10.1136/bmj.330.7495.843 15817555 PMC556084

[14] Herdman M, Gudex C, Lloyd A, et al Development and preliminary testing of the new five-level version of EQ-5D (EQ-5D-5L). Qual Life Res 2011;20:1727–36. 10.1007/s11136-011-9903-x 21479777 PMC3220807

[15] Chow S, Shao J, Wang H Sample size calculation in clinical research. New York: Taylor & Francis, 2003.

[16] Nolan CM, Longworth L, Lord J, et al The EQ-5D-5L health status questionnaire in COPD: validity, responsiveness and minimum important difference. Thorax 2016;71:493–500. 10.1136/thoraxjnl-2015-207782 27030578 PMC4893131

[17] van Hout B, Janssen MF, Feng YS, et al Interim scoring for the EQ-5D-5L: mapping the EQ-5D-5L to EQ-5D-3L value sets. Value Health 2012;15:708–15. 10.1016/j.jval.2012.02.008 22867780

[18] Manca A, Hawkins N, Sculpher MJ Estimating mean QALYs in trial-based cost-effectiveness analysis: the importance of controlling for baseline utility. Health Econ 2005;14:487–96. 10.1002/hec.944 15497198

[19] Rubin DB Multiple imputation after 18+ years. J Am Stat Assoc 1996;91:473–89. 10.1080/01621459.1996.10476908

[20] Kon SS, Canavan JL, Jones SE, et al Minimum clinically important difference for the COPD Assessment Test: a prospective analysis. Lancet Respir Med 2014;2:195–203. 10.1016/S2213-2600(14)70001-3 24621681

[21] Puhan MA, Frey M, Büchi S, et al The minimal important difference of the hospital anxiety and depression scale in patients with chronic obstructive pulmonary disease. Health Qual Life Outcomes 2008;6:46 10.1186/1477-7525-6-46 18597689 PMC2459149

[22] Treasure T, MacRae KD Minimisation: the platinum standard for trials?. Randomisation doesn’t guarantee similarity of groups; minimisation does. BMJ 1998;317:362–3. 9694748 10.1136/bmj.317.7155.362 PMC1113668

[23] Rehal S, Morris TP, Fielding K, et al Non-inferiority trials: are they inferior? A systematic review of reporting in major medical journals. BMJ Open 2016;6:e012594 10.1136/bmjopen-2016-012594 PMC5073571 27855102

[24] Jeppesen E, Brurberg KG, Vist GE, et al Hospital at home for acute exacerbations of chronic obstructive pulmonary disease. Cochrane Database Syst Rev 2012:CD003573 10.1002/14651858.CD003573.pub2 22592692 PMC11622732

[25] Skwarska E, Cohen G, Skwarski KM, et al Randomized controlled trial of supported discharge in patients with exacerbations of chronic obstructive pulmonary disease. Thorax 2000;55:907–12. 10.1136/thorax.55.11.907 11050258 PMC1745644

[26] Aimonino Ricauda N, Tibaldi V, Leff B, et al Substitutive "hospital at home" versus inpatient care for elderly patients with exacerbations of chronic obstructive pulmonary disease: a prospective randomized, controlled trial. J Am Geriatr Soc 2008;56:493–500. 10.1111/j.1532-5415.2007.01562.x 18179503

[27] Nicholson C, Bowler S, Jackson C, et al Cost comparison of hospital- and home-based treatment models for acute chronic obstructive pulmonary disease. Aust Health Rev 2001;24:181–7. 10.1071/AH010181 11842709

[28] Goossens LM, Utens CM, Smeenk FW, et al Cost-effectiveness of early assisted discharge for COPD exacerbations in The Netherlands. Value Health 2013;16:517–28. 10.1016/j.jval.2013.01.010 23796285

[29] Cotton MM, Bucknall CE, Dagg KD, et al Early discharge for patients with exacerbations of chronic obstructive pulmonary disease: a randomized controlled trial. Thorax 2000;55:902–6. 10.1136/thorax.55.11.902 11050257 PMC1745631

[30] Davies L, Wilkinson M, Bonner S, et al “Hospital at home” versus hospital care in patients with exacerbations of chronic obstructive pulmonary disease: prospective randomised controlled trial. BMJ 2000;321:1265–8. 10.1136/bmj.321.7271.1265 11082090 PMC27532

[31] Ojoo JC, Moon T, McGlone S, et al Patients’ and carers’ preferences in two models of care for acute exacerbations of COPD: results of a randomised controlled trial. Thorax 2002;57:167–9. 10.1136/thorax.57.2.167 11828049 PMC1746235

[32] Nissen I, Jensen MS Randomised controlled trial of nurse-supported discharge of patients with exacerbation of chronic obstructive pulmonary disease [Danish] Sygeplejeassisteret hjemmebehandling af eksacerbation i kronisk obstruktiv lungesygdom. Ugeskrift for Laeger 2007;169:2220–3. 17592691

[33] Utens CM, Goossens LM, Smeenk FW, et al Early assisted discharge with generic community nursing for chronic obstructive pulmonary disease exacerbations: results of a randomised controlled trial. BMJ Open 2012;2:e001684 10.1136/bmjopen-2012-001684 PMC3488726 23075570

