# Supplementary material for: Home treatment of COPD exacerbation selected by DECAF score: a non-inferiority, randomised controlled trial and economic evaluation
Source: Thorax. 2018 Apr 21;73(8):713–22. doi: 10.1136/thoraxjnl-2017-211197 (PMC6204956; doi:10.1136/thoraxjnl-2017-211197)
Supplement: Supplementary file 1 [file thoraxjnl-2017-211197supp001.pdf]

AECOPD First Review Hospital at Home

Northumbria Healthcare NHS
NHS Foundation Trust

Hospital: NSECH ☐ NTGH ☐ WGH ☐

Date: ___________________

Time: ___________________

First Name: ____________________ Surname: ____________________

Trust No:___________________________ DOB: ___________________

NHS number: ____

Address: ____

**Respiratory problems** Note duration and severity
Breathlessness
Cough
Sputum Purulence
Sputum Volume
Wheeze
Other

**Performance status** Current exercise tolerance
Exercise tolerance on a good day in last 3 months
Is the patient **currently** independent in: **Washing YES / NO Dressing YES / NO** Toileting YES / NO
Feeding YES / NO Cooking YES / NO
Other care needs:

**Other symptoms or problems raising concerns**

**Examination:**	RR	SpO2	FiO2 room air/ 24%/ 28%/ 35% / other		BM
HR	BP	Temp	A V P U / GCS	BMI	
Chest examination					
Dependent oedema: None☐ Ankle☐ Mid-tibial☐ *knee☐ *mid-thigh☐ *sacral☐ *Fasting daily weight ______Kg					

Signature:_________________ Print:_____________________ Grade:___________ ID (NMC/GMC) ________________

GENERAL MEDICINE

AECOPD First Review Hospital at Home

Northumbria Healthcare NHS
NHS Foundation Trust

**Problem list and diagnosis**			
DECAF score		Exacerbation frequency/ year	
Ex-smoker☐ Current smoker☐	Pack years	Industrial exposure	
Last sputum culture	Date		FEV1% predicted
**Acute Treatment** Antibiotic First line		Second line	
Sputum sent Y / N Date sent		Result	
Nebulised bronchodilators YES / NO	Maintenance Inhalers changed YES / NO		
Prednisolone day	Any side effects? (check BMs)		
Current diuretic: None ☐ Drug (dose):			
Diuretic prior to admission: None ☐ Drug (dose):			
Oxygen YES / NO If yes, Venturi mask (most patients) ☐	FiO2: %;	Nasal Cannulae ☐ flow rate:	l/min
Target sats 88-92%			
Carbocisteine YES / NO Tinzaparin YES / NO Chest physio YES / NO			
Other			
**Chronic Treatment** Annual 'flu vaccine YES / NO Pneumococcal vaccine YES / NO			
Inhaler technique adequate YES / NO If no, consider new inhaler:			
Unmet psychological need YES / NO			
HADS score A / D	Referral to health psychology YES / NO		
Increase in care package required YES / NO	Adult social care contacted YES / NO	OT contacted YES / NO	
Unmet nutritional needs YES / NO	Referral to dietician YES / NO		
Exacerbation recognition and self-management education delivered YES / NO			
Importance of activity and pulmonary rehab discussed YES / NO			
Smoking cessation offered YES / NO / Not applicable		NRT prescribed YES / NO / Not applicable	
Other			
Provisional discharge date			

Signature:_________________ Print:_____________________ Grade:____________ ID (NMC/GMC) __________________
