# Supplementary material for: Home treatment of COPD exacerbation selected by DECAF score: a non-inferiority, randomised controlled trial and economic evaluation
Source: Thorax. 2018 Apr 21;73(8):713–22. doi: 10.1136/thoraxjnl-2017-211197 (PMC6204956; doi:10.1136/thoraxjnl-2017-211197)
Supplement: Supplementary file 2 [file thoraxjnl-2017-211197supp002.pdf]

AECOPD Daily Review Hospital at Home

Northumbria Healthcare NHS
NHS Foundation Trust

Location: Home / Hospital ________________	First Name: __________________ Surname: ___________________
Date/Time: __________________ __________	Trust No:__________________________ DOB: __________________
Days in Hospital:__________________________	NHS number: ____
Days at Home: ___________________________	Address: ____

**Respiratory problems** Note duration and severity at first, then change over the last 24 hours subsequently
Breathlessness
Cough
Sputum Purulence
Sputum Volume
Wheeze
Other
**Performance status** Change over last 24 hours: Better / Similar / Worse
Comments
Difficulty with usual activities YES / NO Describe
**Other symptoms or problems raising concerns**
**Examination:** RR SpO2 FiO2 room air/ 24%/ 28%/ 35% / other BM
HR BP Temp A V P U / GCS Fasting daily weight (Kg)
Chest examination
Dependent oedema: None☐ Ankle☐ Mid-tibial☐ *knee☐ *mid-thigh☐ *sacral☐ *Fasting daily weight ______Kg

Signature:_________________ Print:_____________________ Grade:____________ ID (NMC/GMC) __________________

GENERAL MEDICINE

AECOPD Daily Review Hospital at Home

Northumbria Healthcare NHS
NHS Foundation Trust

Problem list and diagnosis

Any new diagnosis

ABG done (do if drop in SpO2) YES / NO Bloods done YES / NO

Acute Treatment Antibiotic Day Change if no improvement after 48 hours

Sputum sent Y / N Date sent Result MRSA status +ve / -ve

Nebulised bronchodilators YES / NO

Prednisolone day Any side effects? (check BMs)

Weight change in 24 hours

Current diuretic: None☐ Drug (dose)

Diuretic prior to admission: None☐ Drug (dose)

Oxygen YES / NO If yes, Venturi mask (most patients)☐ FiO2: %; Nasal Cannulae☐ flow rate: l/min

Target sats 88-92%

Carbocisteine YES / NO Tinzaparin YES / NO Physio review YES / NO (all patients day 3) Home exercise YES / NO

Other

Chronic Treatment Inhaler technique adequate YES / NO If persistently inadequate, consider changing inhaler

Referral required: Psychology YES / NO Nutrition YES / NO Change in care package YES / NO

Describe issues and action

Exacerbation recognition and self-management education delivered (including smoking cessation) YES / NO

Importance of activity and pulmonary rehab discussed YES / NO

Consider azithromycin, bone protection near time of discharge from Hospital at Home

Other

Provisional discharge date Follow up: Clinic Doctor / Nurse (doctor if CXR consolidation)
COPD care Bundle complete YES / NO SPUDS YES / NO

Signature:_______________ Print:_________________ Grade:__________ ID (NMC/GMC) _______________
