# Supplementary material for: Home treatment of COPD exacerbation selected by DECAF score: a non-inferiority, randomised controlled trial and economic evaluation
Source: Thorax. 2018 Apr 21;73(8):713–22. doi: 10.1136/thoraxjnl-2017-211197 (PMC6204956; doi:10.1136/thoraxjnl-2017-211197)
Supplement: Supplementary file 3 [file thoraxjnl-2017-211197supp003.pdf]

Hospital at Home Manual

Dr Stephen Bourke
Dr John Steer
Dr Carlos Echevarria
Mrs Elizabeth Norman

NORTHUMBRIA HEALTHCARE NHS FOUNDATION TRUST

Contents

Hospital at Home selected by low risk DECAF score 3
Useful numbers: 3
What support is offered within Hospital at Home? 4
Return to Hospital 4
Identifying eligible patients 4
Selection criteria 4
DECAF score 4
Management pathway 5
In hospital planning for Hospital at Home 5
RSN review 5
AECOPD proforma 6
Assessment of needs 6
Hospital at Home COPD return home checklist 6
Patients' medical notes and admission file 6
Medication prior to return home 6
Procedure for Completion of Drug Chart for Patients receiving HAH 7
Organising transport for return home 9
Maintaining the Patient at Home 9
RSN review 9
AECOPD proforma 10
Kardex 10
Oxygen 10
Blood monitoring 10
Occupational therapy 10
Physiotherapy 10
Psychological Therapy 11
Adult Social Care 11
Out of hours calls 11
Return to Hospital 11
Identifying patients who need to return to hospital 11
Ambulance transfer from Hospital at Home to hospital 11
Organising the ambulance for return to hospital 12
MRSA and C. Diff. 12
Discharge from Hospital at Home 12

Appendix 14
Hospital at Home Return Home Checklist 14
Equipment check for Home visits 15
COPD Extended Care Bundle 17
Medication Administration Record 19
North East Ambulance Service 20
Consent form for admission notes to return home with the patient 21
COPD re-admission avoidance checklist 22
Working with and Managing Psychological Distress in the Hospital at Home project 23
A. Background 23
B. Levels of help 23
C. Respiratory Clinical Psychology – potential options of help 24

Hospital at Home selected by low risk DECAF score

Chronic Obstructive Pulmonary Disease (COPD) is a common lung disease characterised by progressive breathlessness, cough and phlegm. Acute exacerbations (AECOPD) are episodes, often triggered by infection, during which symptoms deteriorate and are the second commonest reason for hospital admission.

Hospital at home (HAH) manages patients in their own home for a condition that otherwise would require inpatient care. The National Institute for Health and Care Excellence endorse HAH for AECOPD and highlight that selection should be based on prognosis, but acknowledge the (previous) lack of a suitable tool. To address this shortfall, we developed the DECAF score (*Steer, Thorax 2012* and *Echevarria, Thorax 2016*), which accurately predicts survival in patients hospitalised with AECOPD. Of importance, approximately 50% of patients currently admitted to hospital have a low risk of death (1 - 1.4%), thus are potentially suitable for HAH. This is more than twice the proportion of patients included in earlier trials. Our model of HAH includes 24/7 clinical and social support, tailored to the individual patient's needs. The range of healthcare disciplines and level of support available are greater than typically seen in previous trials of HAH or early supported discharge, reflecting the broad selection criteria.

We conducted a Randomised Controlled Trial (RCT) at Northumbria Hospitals to compare HAH to usual inpatient care in patients with AECOPD and low risk DECAF score (supported by a Research for Patient Benefit grant). This model of HAH proved to be safe (no acute deaths), clinically effective (no increase in readmissions), cost-effective and was preferred by 90% of patients. To explore factors influencing wider implementation of this model of care, patients, carers, clinicians and hospital managers were interviewed. Patients valued the availability of home comforts, greater independence and continuity of care provided by the HAH specialist team. Positive influences on perceived rate of recovery, sleep quality, mood, convenience for friends and family (particularly grandchildren visiting) and carer burden were also reported. A few patients were concerned about being alone, particularly at night (a 9pm phone call was valued), or professionals visiting their home. In the early phase of the trial, clinician concerns occasionally delayed return home. Nurses cited greater workload and responsibility, but providing HAH was viewed positively. Operational concerns included keeping medical records in a patient's home and inability to capture activity within current payment systems.

Useful numbers:

Respiratory Specialist Nurse for Hospital at Home: XXXX
Respiratory Consultant (NSECH): XXXX
HOOF: XXXX
Adult Social Care, Northumbria patients (Wansbeck General Hospital): XXXX
Adult Social Care, North Tyneside General Hospital: XXXX
Escalation plan number NE Ambulance Service: XXXX
Consultant clinical psychologist: XXXX

What support is offered within Hospital at Home?

Prior to return home under HAH, patients will be reviewed by a respiratory consultant and respiratory specialist nurse (RSN) to confirm the diagnosis, eligibility and both acute and chronic disease management. The HAH package of care is tailored to the individual patient's needs. The patient will be reviewed by a physiotherapist regarding breathing control, sputum clearance, home exercise programme and subsequent early pulmonary rehabilitation. Access to a pharmacist, occupational therapist and same day short term social support is available. Both a nebuliser and temporary controlled oxygen therapy will be supplied, with instruction on use at home, if required. The specialist nurse will accompany the patient during return home and oversee clinical management at home.

Patients receiving HAH are seen at least once daily by a RSN and undergo monitoring of their respiratory rate, oxygen saturation, heart rate, blood pressure, temperature and, if they have significant dependent oedema, daily weight. They have access to most of the medical treatments available in hospital, including intravenous therapy, but excluding acute non-invasive ventilation. The RSNs provide 24-hour telephone support throughout the duration of HAH, with consultant support.

During HAH, the specialist respiratory team retain clinical responsibility for the patient. At the end of Hospital at Home the patient will be "discharged" as if they had been in hospital.

Return to Hospital

"Return to Hospital" is the term used to describe a patient returning to hospital during a period of HAH and is regarded as an increase in the level of care, not a readmission. The patient remains under the care of the specialist team throughout the HAH period, and will contact the RSN directly if they are concerned. The RSN may provide reassurance or arrange a home visit and/or return to hospital, in liaison with the on-call respiratory consultant. "Readmission" describes the patient returning to hospital after they have been discharged from HAH.

Identifying eligible patients

Patients with an exacerbation of COPD triaged for hospital admission who are low risk (DECAF 0 or 1) should be considered for Hospital at Home treatment. Patients will be primarily identified by the RSNs screening and assessing new respiratory admissions.

Selection criteria

- Primary diagnosis of AECOPD
- DECAF score 0 or 1
- No other acute condition which necessitates hospital admission
- Absence of acute confusion precluding discharge

DECAF score

Dyspnoea: please remember to ask the patient about breathlessness on a good day within the last three months, not on admission.

Acidaemia: if an arterial blood gas has not been performed, provided the patient's SpO_2 is 92% or greater breathing room air, it is highly unlikely that the patient's pH < 7.30 (threshold required to score). If a venous blood gas pH is not acidaema, the arterial pH cannot be acidaemia (arterial pH = venous pH + 0.03).

DECAF Score		Circle
D*	eMRC**D** 5a (Too breathless to leave the house unassisted but independent in washing and/ or dressing)<br>eMRC**D** 5b (Too breathless to leave the house unassisted and requires help with washing and dressing)	1<br>2
E	**E**osinopenia (eosinophils < 0.05 x10^9/L)	1
C	**C**onsolidation	1
A†	Moderate or severe **A**cidaemia (pH < 7.3)	1
F	Atrial **F**ibrillation (including history of paroxysmal AF)	1
	**Total:**	

***** Ask about breathlessness on a good day within the last 3 months, not during an exacerbation/on admission.
† If a blood gas has not been performed, provided oxygen saturation breathing room air is > 92%, acidaemia can be assumed not to score.
In-hospital mortality risk: DECAF 0-1 (low) = 1 - 1.4%; DECAF 2 (intermediate) = 5.4 – 8.4%; DECAF 3+ (high) = 21.4 – 34.7%.

Management pathway

Patients with a low risk DECAF score managed in hospital are unlikely to require an escalation in care. When this does occur, the patient is usually hypercapnic on admission and their condition typically deteriorates within the first 24 hours. Patients who are acidaemic on admission but otherwise low risk by DECAF remain at low risk (Echevarria Thorax 2016).

The timing of return home under hospital at home is determined by the admission arterial blood gas.

- Normal $PaCO_2$ or SpO_2 >92% breathing room air & ABG considered clinically unnecessary = return home as soon as possible (most patients).
- Hypercapnia, normal pH = return home if not deteriorating at 24hrs.
- Hypercapnia and pH < 7.35 = return home within 24 hrs of resolution of acidaemia, and discontinuation of acute NIV if provided.

In hospital planning for Hospital at Home

RSN review

The RSNs screen and assess new admissions to the acute respiratory admissions unit. Some eligible patients may have already been identified by medical staff, and will be highlighted to the RSN.

When a suitable patient is identified for HAH, the RSN will ensure all required clinical assessments have been performed and that adequate support is in place. This may involve delegating tasks to ward staff. The RSN will facilitate return home. For HAH to work effectively, flexibility and clear communication is required.

AECOPD proforma

The AECOPD proforma includes an initial assessment sheet, which should be completed by the RSN on the day the patient returns home, and daily review sheets to be completed on subsequent days during HAH.

Assessment of needs

Following review by the RSN and respiratory consultant, a management plan will be put in place including: 1) acute management of this exacerbation; 2) review and optimisation of long-term COPD management; and 3) assessment of home circumstances. This may involve reviewing patients on a non-respiratory ward. On occasion, if the consultant is not available, the medical review may be performed by a specialist respiratory registrar. The RSN and ward staff will identify the patient's social support needs. Adult social support should be contacted immediately if same day support is required.

Hospital at Home COPD return home checklist

This will be completed prior to the patient returning home, and is a simple checklist to ensure all the practical aspects of discharge have been met.

Patients' medical notes and admission file

The AECOPD proforma should be filed in red admission file with the admission clerking, nursing notes, drug kardex and observation chart. The red admission file will follow the patient home and remain with them throughout the HAH period of care. Prior to return home, a photocopy of the current admission documentation in the red file should be made and filed in volume 1 of the medical notes (retained on the respiratory ward for the duration of HAH to ensure ease of access if required). There is a consent form related to retention of the red file in the patient's home during HAH. The RSN will ensure this is signed by the patient.

If the patient returns to hospital during HAH the red file **must** accompany the patient. Whilst such patients require a thorough review, they will not need a new clerking or new drug Kardex. On discharge from HAH, the red file should be returned to the respiratory ward and the original complete admission documents filed in volume 1 of the patients notes, replacing the earlier photocopy.

Medication prior to return home

Not all RSNs have completed the prescribing course. All medication, including anticipated medication, should be prescribed before the patient leaves hospital. See below "Procedure for Completion of Drug Chart for Patients receiving HAH" below for further information.

Antibiotic protocol for Hospital at Home

No allergies / History of intolerance

1. Not previously treated, or treated with Amoxicillin: First line- Doxycycline; Second line- Co-amoxiclav.

2. Previously treated with Doxycycline: First line- Co-amoxiclav; second line- Levofloxacin 500mg bd.

Intolerant of Doxycycline

First line- Co-amoxiclav; second line- Levofloxacin 500 mg bd.

Intolerant of Penicillin

First line- Doxycycline; second line- Levofloxacin 500 mg bd.

If a patient is colonised with Haemophilus Influenza (recurrent positive cultures), an extended course of co-amoxiclav of 10-14 days is appropriate.

If pseudomonas is cultured, consider oral ciprofloxacin or intravenous antibiotics depending on sensitivities. Discuss dose and duration with a consultant.

Proton Pump Inhibitors

Proton pump inhibitors are (PPI) commonly stopped in patients when starting antibiotics due to the risk of Clostridia Difficile (C diff). It is important to balance the risks.

GI bleed within 12 months score 2; GI bleed > 12 months score 1; Female score 1; Co-prescription antiplatelet therapy, NSAIDS or SSRIs score 1; Co-prescription oral anticoagulant score 1; Chronic renal disease GFR < 60 score 1; BMI < 19 score 1.
Total risk score:

- 2+ continue PPI
- 1 switch to H2 antagonist for the duration of antibiotic therapy plus 7 days
- 0 withhold PPI for the duration of antibiotic therapy plus 7 days
- No existing indication for PPI on review – discontinue.

Procedure for Completion of Drug Chart for Patients receiving HAH

Patients suitable for HAH treatment will already have had their inpatient drug chart completed with the necessary medicines to treat the acute exacerbation. Prior to the patient being sent home the RSN should ensure that the following medication is prescribed:

Regular medication:

a. Tinzaparin prophylaxis if deemed necessary on VTE assessment – discontinue once the patient achieves their normal level of mobility.
b. Salbutamol 2.5mg nebules four times a day – with a note to be reviewed 48 hours post admission.
c. Ipratropium Bromide 500 microgram nebules four times a day – with a note to be reviewed 48 hours post admission.
d. Prednisolone 30mg once daily for 5 days
e. 1st line antibiotic including stop/ review date – according to antibiotic protocol.
f. Patient’s regular inhalers – if patient on a LAMA place a cross in the administration box for the first 48 hours and a note to withhold whilst on regular ipratropium nebules.
g. Patient’s regular medication – this should be reconciled by the pharmacy team prior to return home. If the patient hasn’t been seen by a member of the pharmacy team (due to

time of admission etc.), please ensure this is reviewed by a doctor. All appropriate medication must be prescribed.

h. Oxygen – if the patient needs oxygen, this must be prescribed on the drug chart.
i. Nicotine patches and short acting nicotine replacement (inhalator, gum or lozenge) if patient a current smoker and willing to have NRT – if patient smokes >20/day start 21mg patch.

As required medication:

a. Salbutamol 2.5mg Nebules for shortness of breath.
b. Ipratropium bromide 500 microgram nebules for shortness of breath.
c. Salbutamol Inhaler (or Terbutaline) – whichever short acting beta2 agonist the patient was using prior to admission.
d. Paracetamol as required.
e. Carbocisteine – 375-750mg up to three times a day if problems clearing sputum (if not already on regular carbocisteine).
f. Sando K tablets – 2 tablets up to three times a day for hypokalaemia. To be commenced only if blood results indicate hypokalaemia.
g. Furosemide – 40-80mg once daily prn if the patient develops worsening dependent oedema. Only to be commenced on discussion with consultant. Occasionally higher dose or intravenous diuretic may be required.
h. OR if not on an ACE-inhibitor, angiotensin receptor blocker or potassium sparing diuretic: Co-amilofruse 5/40 1-2 tablets once daily prn if patient develops worsening dependent oedema. Only to be commenced on discussion with consultant.

Supply of medication:

The RSN should obtain all medication the patient will require during HAH prior to return home. Any medication that the patient takes home with them must either be over-labelled or dispensed from pharmacy: i.e. patients must not go home with unlabelled medication. This includes the patients' regular medication as patients should not have to contact their GP for a prescription during HAH. If required, the RSN will also supply a nebuliser, temporary oxygen concentrator and venturi mask (to achieve target SpO_2 = 88-92%).

Restricted antibiotics (ciprofloxacin and levofloxacin) will be available via the Omnicell cabinets to ensure strict controls are in place.

Discharge from Hospital at Home:

When the patient is discharged from HAH the patient should receive a copy of their discharge letter and the nursing staff must ensure that the patient has sufficient supplies of medication. If a rescue pack is considered appropriate, this must also be included in the discharge letter and supplied to the patient with written and verbal information.

Overlabelled Medication Available Hospital at Home:

Anoro (1 inhaler)
Amoxicillin 500mg capsules (box of 21)
Carbocisteine 375 mg capsules (box of 120)
Co-amilofruse 5/40 tablets (box of 28)
Co-amoxiclav 625mg tablets (box of 21)
Ciprofloxacin 250mg tablets
DuoResp 320/9 (1 inhaler)
Doxycycline 100mg capsules (box of 8)
Furosemide 40mg tablets (box of 28)
Ipratropium 500microgram Nebules (box of 20)
Levofloxacin 500mg tablets
Nicotine 21mg patches (box of 7 patches)
Prednisolone 5mg tablets – (box of 28 for acute course and 42 for rescue pack)
Salbutamol 100 microgram/puff inhaler (1 inhaler)
Salbutamol 2.5mg Nebules (box of 20)
Sando K tablets (tube of 20)
Seebri (1 inhaler)
Tiotropium 18 microgram Inhaler (1 inhaler)
Ultibro (1 inhaler)

Procedure for Completion of Drug Chart written by: Nicola Harker, Senior Clinical Pharmacist.

Organising transport for return home

Patients who do not require controlled oxygen therapy or other assistance during transfer home may use private transport. Plans for transfer home will be reviewed by the RSN. If an ambulance is required the RSN should contact Ambulance Control and request an urgent ambulance (1-2 hour response). The RSN will review return home to help establish the patient in their own home.

A patient should be flagged up to the NEAS (North East Ambulance Service) by sending a referral form (see appendix) to the following email address: XXXXXXX The patient can be removed once they are discharged, or will automatically come off the system after 2 weeks.

Maintaining the Patient at Home

RSN review

The patient will have been reviewed by a RSN prior to returning home. Where possible, the RSN for HAH will accompany the patient home or, if not feasible, visit them shortly after return home. A detailed handover of HAH patients must occur when one RSN takes over from another on the rota. The RSN will have a standard set of equipment that they take to see the patient (see Hospital at Home equipment checklist).

The patient will be reviewed daily during HAH, seven days per week. As well as nurse visits, patients will be routinely contacted by telephone in the evening. In particular, patients living alone often value an evening phone call.

AECOPD proforma

A new daily review sheet should be completed each day. This includes prompts to: review the patient's symptoms and social support needs; perform an examination (recording physiological observations); review treatments (including pulmonary rehabilitation) and the need for bloods/ ABGs; and deliver education to the patient and carer.

Kardex

This will be reviewed and updated by the RSN daily. With consent, it is useful to review the patients' medication store to get an impression of the patient's understanding of, and concordance with, their usual medication. This will inform patient education strategies and medication choice.

Smoking Cessation

Smoking cessation is the most effective treatment for COPD. Highlight that smoking not only causes progression of COPD, but also increases the risk of flare ups / exacerbations. Patients who smoke should have nicotine patches and short term NRT prescribed prior to discharge. Patients are more likely to smoke in their own home compared to hospital; be vigilant. If a patient is smoking during HAH this should be documented, and smoking cessation advice and support should be offered in the patient's home.

MRSA eradication

The results of any MRSA swabs will not be available before the patient returns home. If the patient is MRSA positive, MRSA eradication therapy should be started.

Oxygen

If a patient requires temporary controlled oxygen therapy, the RSN will facilitate the transfer of an oxygen concentrator to the patient's home. The oxygen concentrator may require two people for transfer and set-up, however such patients are more likely to need other support, such as HCAs, OT, or physiotherapy. Oxygen should be prescribed and to maintain saturations between 88-92%, with point of care arterial blood gas monitoring if required.

Blood monitoring

Patients will have venous bloods checked at home by the RSN as and when required, for analysis in hospital. Arterial blood gases (ABG) should be analysed in the patient's home using the point of care analyser. If this is unavailable, the ABG sample should be taking just before leaving the patient's home and transferred to the hospital for immediate analysis on ice: the longer the time to analysis, the greater chance of error.

Occupational therapy

Patients' OT needs will have been identified in the hospital, though new needs may be identified when the RSN returns home with the patient. A key safe will help with access to the house for those patients that are unable to answer the door independently. Key safes will be stored with both the RSNs and with OT. There is an array of available equipment to help patients at home to sit upright in bed, and OTs will choose the best option based on their assessment of the patient.

Physiotherapy

The patients will be assessed by a physiotherapist prior to return home or within the first few days of return home. This will include assessment and education on breathing control,

sputum clearance and an individual exercise plan, working towards pulmonary rehabilitation. Where possible, a joint assessment with the RSN will be performed.

Outside of this formal assessment, patients who need assistance with chest clearance will be identified by the RSN, who will contact the physiotherapist to provide additional support. All patients will be offered pulmonary rehabilitation, to commence within 4 weeks of discharge. Pulmonary rehabilitation is one of the most successful treatments for COPD, and patients may be more receptive to this intervention when education is provided to them and their family in the patient's home.

Psychological Therapy

XXXXXX should be contacted if it is felt that patients could benefit from psychological therapy. Please see appendix "Working with and managing psychological distress in the Hospital at Home project" for further information.

Adult Social Care

Patients who have care needs will be seen by HealthCare Assistants with basic training in COPD. RSN should coincide their visits with HCA visits where possible as this will be helpful, and also an opportunity to deliver additional training and support.

Out of hours calls

Patients will be able to phone the RSN on call for HAH at any time of the day. Based on our experience, the call volume is expected to be low. A routine evening phone call can address any issues, and help prioritise morning visits if there is more than one patient in Hospital at Home, which may minimise overnight calls.

Return to Hospital

Identifying patients who need to return to hospital

The decision to have a patient return to hospital will be made by the RSN. Where possible, the RSN should discuss the patient with a respiratory consultant, and have the results of up-to-date bloods and arterial blood gases available to inform the decision. In some instances, it may be unclear if the patient needs to return to stay in hospital: such a patient can be brought back to hospital for a chest x-ray and medical review, and could return home if sufficiently well. Ideally, this should be carefully co-ordinated to allow a timely review once the chest x-ray, bloods and ABG have been performed.

Ambulance transfer from Hospital at Home to hospital

A minority of patients receiving Hospital at Home may need to return to hospital. NEAS will be informed of, and maintain a record of, all patients receiving Hospital at Home.

All patients will have **medical notes (red file) and a drug Kardex** at home, which **must return to hospital** with the patient. If oxygen is required it should be delivered by Venturi mask (target saturations 88-92%).

Patients returning to hospital via this route will bypass Accident and Emergency and go directly to the acute respiratory ward NSECH.

Organising the ambulance for return to hospital

1) The patient contacts Respiratory Special Nurse (RSN); outside of office hours, the nurse will either:
    a) Offer phone advice and visit the patient in the morning
    b) Organise return to hospital
2) If return to hospital is required, the RSN will contact the bed manager to arrange a bed on the acute respiratory ward. Patients will bypass Accident and Emergency.
3) The RSN will phone for an ambulance, indicating the level of urgency and the patient's destination.

MRSA and C. Diff.

If a patient has watery stools (stool type 7 on the Bristol Stool Scale) or is found to be MRSA positive, then the patient will need to be isolated when they return to hospital. The bed manager should be informed as early as possible.

Discharge from Hospital at Home

The COPD care bundle should be completed for all patients who are discharged from Hospital at Home, as occurs in hospital. A copy will be sent by the RSN to hospital pharmacist. The RSN will liaise with pharmacy to let them know the patient has been discharged so they can perform a one week follow-up phone call (including review of education regarding rescue medication).

The decision to discharge a patient from Hospital at Home will be made by the RSN in liaison with the respiratory consultant. It is expected that the average duration of HAH will be similar to the length of hospital stay for an acute exacerbation of COPD managed as an inpatient (4-5 days).

The criteria below can be used to help guide when a patient is ready for discharge. All patients will vary, and the patient's baseline status must also be considered.

- Symptoms improving (breathlessness, sputum)
- Oxygen saturations >88% on room air, or if on LTOT usual oxygen requirements
- Pulse less than 110
- Respiratory rate less than 25
- Systolic blood pressure greater than 90mmHg
- Apyrexial for greater than 24 hours
- Off nebulisers for greater than 24 hours
- Mobility adequate
- Social support adequate and in place

The discharge from HAH date must be clearly documented in the notes and a discharge letter will be dictated on G2 by the RSN (with copies for the patient, their GP and the

medical notes). If the patient needs a 2-week supply of medication and an emergency pack this will need to be prescribed.

The red file will be returned to the hospital by the RSN. It should be given to the ward clerk on the ward from which the patient returned home. Patients in HAH remain eligible for all treatments and services that are available for those being discharged from hospital, such as Supported Pulmonary Discharge (NTGH) and community matron review (WGH). Patients should have 6 week follow-up in respiratory clinic, either with a respiratory nurse or doctor. The patient should be seen by a doctor of they have consolidation on their chest x-ray.

Appendix

Hospital at Home Return Home Checklist

	Required	Done
Patient reviewed by consultant (resp or gen med)		
Patient reviewed by respiratory consultant or registrar		
All jobs addressed from consultant ward rounds		
Discharge medications organised		
Family/carer informed patient is returning home		
RSN phone number given to patient and family/ carer		
Letter faxed to GP + filed in notes		
Admission details photocopied; copy left in hospital notes		
Consent from for admission notes to return home signed		
Inform North East Ambulance service		
Inform Northern Doctors		
Dementia CQUIN target completed		
VTE (DVT) CQUIN target completed		
Oxygen concentrator organised, forms signed	Yes / No	
OT organised	Yes / No	
Physio organised *	Yes / No	
Adult social care organised	Yes / No	
Ambulance organised	Yes / No	
- Patient flagged on NE ambulance service system		
Equipment prepared for home visit		

*All patients should receive early pulmonary rehabilitation

Equipment check for Home visits

Required?	YES	NO
Medication pack		
Salbutamol nebules		
Ipratropium bromide nebules		
Amoxillin (low dose and high dose)		
Co-amoxiclav		
Doxycycline		
Ciprofloxacin		
Prednisolone		
Furosemide		
Co-amilofruse		
Tinzaparin- 3,500 and 2,500 units		
Nebuliser machine		
Cleaning equipment		
Masks		
Tubing		
Oxygen		
Tubing		
Venturi masks 24 28 35		
Investigations- routine bloods		
Urea and electrolytes blood vials		
Full blood count vials		
Clotting test blood vials		
Needles		
Syringes		
Sharps box		
Sterile skin prep		
Gloves		
Handwash		
Tourniquet		
Cotton wool/ gauze and tape		
Sputum pots		
Arterial Blood Gases		
Arterial Blood Gas syringes		
ABG point of care machine		
ABG machine cartridges		
Ice bag (if ABG machine unavailable)		
Observations		
Oxygen saturations (check working)		

Blood pressure- automatic		
Blood pressure- manual		
Stethoscope		
Temperature		
Scales		
Timer (resp rate)		
BMs		
Telehealth equipment		

Northumbria Healthcare NHS
NHS foundation Trust

COPD Extended Care Bundle

Northumbria Healthcare NHS
NHS Foundation Trust

COPD EXTENDED CARE BUNDLE

Please complete for *all* patients admitted with an *acute exacerbation of COPD*

On discharge: Fax to Resp nurse specialists on ext 4141 and copy to pharmacist

Hospital site NSECH NTGH WGH HGH
Ward number.....................................

Attach patient ID label
Patient name:
Date of birth:
Hospital number:
Patient tel. no:

1. DECAF Score (eMRCD score guidance overleaf)

	Variable	Score
1.	eMRCD5a (Too breathless to leave the house unassisted but independent in washing and/ or dressing)	1
	eMRCD 5b (Too breathless to leave the house unassisted and requires assistance in both washing and dressing)	2
2.	Eosinopenia (eosinophils <0.05 x10⁹/L)	1
3.	CXR consolidation	1
4.	Moderate or severe acidaemia (pH<7.3)	1
5.	Atrial fibrillation (including history of paroxysmal AF)	1
	DECAF Score: =	

2. Smoking cessation

Does the patient smoke?	Yes * / No	
* If current smoker	Do they want to stop	Yes / No
	Prescribed NRT in hospital?<br>Contact details for smoking cessation given	Yes / No / Declined<br>Yes / No / Declined

3. Inhaler technique

Satisfactory?	Yes / No*
	* If not, confirm that the patient has been provided with a device they can use: ☐

4. Rescue medications

Prescribe	Prednisolone 30mg od for 5 days and an antibiotic (instructions overleaf)
Antibiotic prescribed:	

Pharmacist to complete 2, 3 & 4. Name: .... Sign: .... Date:................

5. Pulmonary Rehabilitation

Previous pulmonary rehabilitation?	Yes* / No	* If yes, when?
Referred for pulmonary rehabilitation?	Yes / No / Declined / Not appropriate	

6. BMI

<18.5	GP to refer to dietician re supplements	☐
>30	GP to promote weight reduction, consider dietician referral	☐

7. Spirometry

Pre-admission spirometry in hospital records?: Yes* / No Inpatient spirometry?: Yes* / No

* FEV_1 .... (% predicted) (F)VC .... FEV_1/(F)VC ratio .... Date ....

Post discharge spirometry (circle) At hospital review? GP to arrange?

Faxed to 4141 ☐

September 2015 —Version 3.1

Northumbria Healthcare NHS
NHS Foundation Trust

8. Extended MRC Dyspnoea Score (instructions below)	
"In the **past 3 months**, when you were **feeling at your best**, which of the following statements best describes your level of breathlessness?" (please circle)	
Only breathless on strenuous exertion	1
Breathless hurrying or walking up a slight hill	2
Walks slower than contemporaries, or stops after walking on the level for 15 minutes	3
Stops for breath after walking about 100 yards, or for a few minutes, on the level	4
Too breathless to leave the house unassisted but independent in washing and / or dressing	5a
Too breathless to leave the house unassisted and requires help with washing and dressing	5b

9. Follow Up			
Consultant ☐	Respiratory Specialist Nurse ☐	SPUDS (NTGH only) ☐	Primary Care ☐
Out-patient clinic:	Clinician name	Date and time	

Dr / Nurse to complete 1, 5, 6,7,8 & 9. Name: ....**Sign:**.........................**Date:**........

Additional Information

Antibiotic Choice

First line: **Doxycycline** 200mg oral od for 1 day , then 100mg od for 4 days

Second line: **Co-amoxiclav** oral 625mg tds for 5 days

Third Line: **Check culture & sensitivity. Discuss with clinician.**

Choice depends upon recent antibiotic use, history of intolerance or allergy and culture and sensitivity results (choose narrowest spectrum antibiotic: e.g. if sensitive to amoxicillin px amoxicillin).

eMRCD Scale Guidance Notes:

- Remember that you are asking the patient about their level of breathlessness **on a good day** over the preceding 3 months, **not breathlessness during an exacerbation / on admission**.
- A patient only achieves a higher grade if they are as breathless as defined in that higher grade, for example, if worse than defined in eMRCD 3, but not as bad as eMRCD 4, they remain eMRCD 3.
- A key distinction is between eMRCD 4 and eMRCD 5a/5b: - **only score 5a or 5b if the patient cannot leave the house without assistance.**
  - if a patient can only walk 30 to 40 metres, but can leave the house unassisted, they are eMRCD 4.
  - if a patient can walk 5 or 10 metres, perhaps from their front door to a car, but need a wheelchair otherwise, they require assistance: eMRCD 5a or 5b. (simple walking aids do not constitute assistance)
- If a patient requires assistance in personal washing **and** dressing they are eMRCD 5b. If they only require assistance in washing **or** dressing they are eMRCD 5a. Remember to ask about putting on shoes and socks.
- If patients are limited for a reason other than breathlessness, score based on their functional limitation.

Faxed to 4141 ☐

Northumbria Healthcare NHS
NHS foundation Trust

Medication Administration Record

Medication support provided by social services: only complete if this service is accessed during HAH.

Sheet ___ of ___

OUTCOME OF RISK ASSESSMENT (i.e. Prompt OR Administer):

Name:	D.o.B.	SWIFT / NHS No:
GP Details:	Chemist Details:	
Allergies:	Start Dates:	

**Medication Details** (including name, strength, dose, route and directions)	**Signature**														
	**Date**														
	Morning														
	Lunch														
	Teatime														
	Evening														
	Morning														
	Lunch														
	Teatime														
	Evening														
	Morning														
	Lunch														
	Teatime														
	Evening														

<u>**NOTE:**</u>
All doses must be signed for, if not given for any reason mark with 'O' and ensure documented in the care plan and reported to Team Supervisor

MAR Chart Completed by..……Date...............................

Checked by.. Date...............................

North East Ambulance Service

This sheet can be found at X:\Respiratory\Hospital at Home

North East Ambulance Service NHS
NHS Foundation Trust

Hospital at Home for COPD Flag Referral

The North East Ambulance Service (NEAS) is able to attach a flag to a patient record to identify those who are being treated in Hospital at Home for COPD under North Tyneside and Wansbeck General Hospital. Whilst under Hospital at Home, patients who return to hospital will bypass accident and emergency and go straight to the medical admissions unit/ emergency care unit.

To inform the NEAS of a new patient, to update or remove an existing patient's flag please complete the form below.

Patient Status	New: ☐	Existing: ☐	Remove: ☐
Surname			
Forename			
DOB			
Telephone No			
NHS Number			
Address (inc. postcode)			
GP Name and Practice			
Documentation in Place:	All patients at home will have their medical records (**red file and drug kardex**) which must return to hospital with the patient. Patients will be in Hospital at Home for 1 to 2 weeks, during which they time they will be seen at home regularly by hospital staff. They will have 24 hour a day contact to a Respiratory Specialist Nurse, who can arrange return to hospital. In the unlikely event that the patient contacts the ambulance service directly, they ambulance crew should contact the bed manager before returning the patient directly to the medical admissions unit/ emergency care unit.		
Location of Documents in Property (if known)			
Referrer Name and Contact Details			
Preferred alternative pathways of care should 999 or 111 be contacted (e.g. known to Palliative Care Team and contact details):	Please provide details if available:		
Other Relevant Information (e.g. specific wishes around admission):	Please add the following information with the red flag: "Hospital at Home for COPD patient. If patient returns to hospital please return medical notes (red file and kardex)"		
Patient consents to sharing of information with NEAS (required for flagging): ☐			

Please ensure that if a patient no longer requires their record flagged with NEAS that this form is resent using the 'Remove' option in the patient status field.

Once completed please email (preferred) or fax this form to the following:

Secure email: handover.form@nhs.net or Fax: 0191 430 2081

Please note: This document, when completed, contains patient identifiable information. To email this form securely it must be sent from an nhs.net account.

v1.1, Jan 2014

Consent form for admission notes to return home with the patient

This sheet can be found at X:\Respiratory\Hospital at Home

PATIENT NAME<br>NHS NUMBER<br>DATE OF BIRTH<br>ADDRESS

I ……………………………………………………… consent to my medical notes staying in my home for the duration of my treatment in Hospital at Home. I understand that it is my responsibility to control access to my notes by people not from the hospital, including by family members and friends. I accept that there is a risk that someone may look in my notes without my permission.

I understand that my medical notes are the legal record of the medical assessment and treatment I receive. The attending clinical team will have full access to my records and will return the notes to the hospital at the end of this period of care. Should I become unwell, and need to return to hospital, it is important that these notes are brought back to hospital with me. The notes remain the property of Northumbria Healthcare NHS Foundation Trust.

		DD	MM	YYYY
**Patient (PRINT)**	**Signature**	**Date**		
		DD	MM	YYYY
**Witness (PRINT)**	**Signature**	**Date**		

COPD re-admission avoidance checklist

Intervention	Done
Smoking cessation – advice, NRT, referral	☐
Arrange Long Term Oxygen Therapy assessment if required (pO2 <7.3 kPa; or <8kPa + cor pulmonale* or polycythaemia**)	☐
Pulmonary rehab assessment and referral. Copy discharge letter to physio.	☐
Education to patient and carer including self-management	☐
- Rescue pack issued (check sensitivities from previous sputum)	☐
- Inhaler technique and concordance	☐
Annual 'flu vaccination	☐
Pneumonia vaccination	☐
Azithromycin in recurrent exacerbators (benefit primarily in non-smokers)	☐
Consider supported discharge	☐
Liaise with other members of MDT (e.g. community matron)	☐
Contact number – respiratory outreach service	☐
Follow up appointment	☐
Consider nebuliser assessment for select patients (full trial by protocol)	☐
Discuss patient with supervising consultant pre-discharge, focusing on re-admission avoidance	☐

*Echocardiogram showing right ventricular dysfunction
*Clinical signs of right sided heart failure such as ankle oedema
**Raised haemoglobin and/ or haematocrit

Working with and Managing Psychological Distress in the Hospital at Home project

A. Background

Patient's experiencing an acute exacerbation of COPD will commonly and understandably experience relatively high level of anxiety, low mood (loss of pleasure/ poor sleep etc) and panicky feelings intertwined with increased levels of breathlessness. (*Maurer et al 2008*). However in most cases this could be expected to reduce over time (both towards the end of a 'hospital' episode and during the first several weeks of discharge and rehabilitation) with the resolution of the exacerbation and gradual return to usual day to day functioning. Taking a bio-psycho-social approach to understanding distress is key is helping people.

B. Levels of help

1. Emotional support by front line staff in the hospital at home project (CNS's, physio's etc) is a core requirement in the care of individual patients and their families. Good listening, communication and relationship building skills help patients to feel supported, valued, and understood. Also appropriate psycho-education and information giving will help patients to understand and normalise their distress in the context of their exacerbation.

2. Front line staff can provide advice and support their patients in basic skills to manage distress e.g. breathing control to manage panic/ anxiety, use of distraction, use of relaxation.

3. Good care management plans that actively involve patients (and family/ carers in the home) and are tailored to their specific needs can go a long way to helping patients feel calmer, more in control and hopeful about improvement through an exacerbation.

e.g. – small, gradually increasing mobility goals and help with practice and achieving these as part of rehab. plan through the hospital at home period
- gradually increasing independence in self-care throughout the exacerbation/ hospital at home period
- referral onto SPUDS and community matron support post hospital at home period
- referral onto rehab. at home or in hospital after hospital at home.

4. Be alert to social triggers to a patients distress:

e.g. social isolation, need for external carers etc.

➡ involvement of social services

e.g. other family members anxiety, lack of knowledge/ understanding of COPD or lack of confidence in supporting the person with breathlessness

➡ Talking with and involving partners/ family carers

5. Where distress in relation to COPD and its symptoms remains high and persistent, despite these areas being addressed, or the person is struggling to engage in these areas of help, then referral to respiratory clinical psychology can be considered. if felt to be appropriate, this can be considered during the exacerbation (after initial 3-4 days) or at a 6 week post exacerbation review.

C. Respiratory Clinical Psychology – potential options of help

Respiratory clinical psychology can provide:

1. Staff case consultation, discussion and advice

2. Staff training, support and supervision in their own delivery of basic psychological help and support to distressed patients

3. Patient assessment and advice to contribute to multidisciplinary understanding, planning and management of the patient.

4. If appropriate and the patient is willing to engage in and use a psychological approach to the management of their distress, providing a contracted piece of therapeutic work (typically 6-8 sessions, but tailored to individual need).

5. Ongoing liaison with MDT members involved in the care of the patient.
