# Supplementary material for: Home treatment of COPD exacerbation selected by DECAF score: a non-inferiority, randomised controlled trial and economic evaluation
Source: Thorax. 2018 Apr 21;73(8):713–22. doi: 10.1136/thoraxjnl-2017-211197 (PMC6204956; doi:10.1136/thoraxjnl-2017-211197)
Supplement: Supplementary file 4 [file thoraxjnl-2017-211197supp004.pdf]

Online supplement

Healthcare costs

All costs were adjusted to 2015/ 2016 prices.

Medication

The British National Formulary was used to calculate a unit cost for individual medication doses (table E1). This was multiplied by the number of administrations of each medication over the 90 day period. For inpatient care and Hospital at Home care, all individual doses were recorded on a hospital kardex. It was assumed that all patients received 14 days of their usual medication at discharge from UC or HAH. Long-term medication was cross-referenced with the GP. Out-of-hospital medications were costed on the assumption that the patient took their medication without missing doses (if a patient receives a medication but does not take it, the cost remains). There were 358 unique formulations of medication taken.

Table E1: Unit costs of medication per dose.

Type of medication	Cost (£)	Type of medication	Cost (£)	Type of medication	Cost (£)
Acamprosate Ca E.C. 333mg	0.171	Epilim Chrono MR 500mg	0.291	Olanzapine 2.5mg	0.031
Accrete D3	0.049	Eprosartan 600mg	0.499	Omeprazole 10mg	0.277
Acetylctsteine nebulisers	2.126	Ertapenem 1g iv	31.65	Omeprazole 20mg	0.232
Aciclovir 200mg	0.065	Erythromycin 250mg	0.182	Omeprazole 40mg iv	1.294
Aciclovir 800mg	0.108	Escitalopram 20mg	0.057	Oromorph/oral morphine 10mg/5ml	0.018
Aclidinium bromide	0.477	Esomeprazole 20mg	0.121	Oxybutynin 5mg	0.038
Acitretin 10mg	0.397	Esomeprazole 40mg	0.141	Pantoprazole 40mg	0.048
Adcal D3 400/1.5g	0.026	Etanercept injection 50mg/ml	89.38	Paracetamol dispersible 500mg	0.076
Alendronic acid 70mg	0.230	Felodipine MR 10mg	0.202	Paracetamol iv 1gm	1.133
Alfentanil 500mcg/ml	0.634	Ferrous fumarate 210mg	0.033	Paracetamol oral	0.026
Allopurinol 100mg	0.031	Ferrous sulphate 200mg	0.063	Paracetamol suspension 250mg/5ml	0.010
Amiloride 5mg	0.240	Fexofenadine 120mg	0.105	Paroxetine 20mg	0.066
Aminophylline iv 250mg/10ml	0.650	Finasteride 5mg	0.049	Paroxetine 30mg	0.060
Aminophylline MR 225mg	0.043	Fluconazole 150mg	0.890	Peptac	0.004
Amitriptyline 10mg	0.030	Fluconazole 50mg	0.126	Perindopril 2 mg	0.038
Amitriptyline 25mg	0.031	Fluoxetine 20mg	0.033	Perindopril 4mg	0.046
Amitriptyline 50mg	0.037	Fluoxetine 60mg	0.479	Phenoxymethylpenicillin 250mg	0.039
Amlodipine 10mg	0.031	Folic Acid	0.032	Phosphate enema(standard)	0.031
Amlodipine 5mg	0.029	Fondaparinux 5mg/ml	6.279	Phyllocontin 225mg	0.043
Amoxicillin 1g iv	1.096	Fortisip	1.400	Piroxicam 20mg	0.129

Amoxicillin 250mg	0.057	Fortisip compact drink	2.020	Pramipexole 180mcg	0.041
Amoxicillin 500mg	0.068	Furosemide iv 50mg	0.660	Pravastatin 40mg	0.057
Amoxicillin 500mg iv	0.548	Furosemide 20mg	0.029	Prednisolone 5mg	0.035
Amoxicillin suspension 250mg/5ml	0.012	Furosemide 40mg	0.028	Prednisolone 5mg soluble	1.783
Anusol ointment	0.083	Furosemide iv 20mg/2mls	0.350	Prednisolone Gastro-res 5mg	0.051
Aqueous cream	0.010	Gabapentin 100mg	0.025	Pregabalin 100mg	1.150
Arachis oil enema	37.50	Gabapentin 300mg	0.034	Pregabalin 200mg	1.150
Aspirin 300mg	0.105	Gaviscon susp	0.014	Pregabalin 300mg	1.150
Aspirin 75mg	0.035	Gentamicin iv 80mg/2ml	1.000	Pregabalin 75mg	1.150
Aspirin dispersible 75mg	0.027	Gliclazide 40mg	0.120	Pro-cal shots 30ml	0.024
Aspirin EC 75mg	0.030	Gliclazide 80mg	0.035	Procyclidine	0.127
Atenolol 25mg	0.028	Glycopyrronium inhaler 44mcg	0.917	Prostap 3 DCS	225.7
Atenolol 50mg	0.028	Goserelin implant 10.8mg	235.0	Pulmicort turbohaler 400	0.277
Atorvastatin 10mg	0.037	GTN 5mg iv	14.76	Quetiapine 150mg	0.043
Atorvastatin 20mg	0.043	GTN spray 400mcg	0.017	Quinine Sulphate 200mg	0.067
Atorvastatin 40mg	0.050	Hydrocortisone 100mg iv	0.917	Quinine Sulphate 300mg	0.075
Atorvastatin 80mg	0.086	Hydromol cream	0.044	Ramipril 1.25mg	0.038
Aveeno cream 1%	0.040	Hydromol ointment	0.023	Ramipril 10mg	0.043
Azithromycin 250mg	0.385	Hydroxocobalamin 1mg/ml	2.182	Ramipril 2.5mg	0.037
Beclometasone nasal spray 50mcg	0.011	Hydroxychloroquine 200mg	0.081	Ramipril 5mg	0.036
Bendroflumethiazide 2.5mg	0.026	Hyoscine hydrobromide s/c 400mcg	3.775	Ranitidine 150mg	0.022
Betnovate cream 0.1%	0.132	Hypotonic saline neb 3 or 6%	0.649	Risedronate 35mg	0.248
Biotene oral gel	0.089	Hypromellose eye drops 0.3%	0.109	Rivaroxaban 15mg	1.800
Bisacodyl 5mg	0.035	Ibuprofen 400mg	0.043	Rivaroxaban 20mg	1.800
Bisoprolol 1.25mg	0.037	Ibuprofen Gel 10%	0.049	Ropinirole 500mcg	0.074
Bisoprolol 10mg	0.031	Ibuprofen gel 5%	0.046	Salbutamol evohaler 100mcg	0.008
Bisoprolol 2.5mg	0.034	Imipramine 25mg	0.041	Salbutamol nebules 2.5mg	0.096
Bisoprolol 5mg	0.030	Indapamide 2.5mg	0.051	Salbutamol nebules 5mg	0.191
Budesonide formoterol inhaler	0.275	Instillagel 10ml	0.234	Saline nebs 2.5ml	0.675
Bumetanide 1mg	0.043	Ipratropium nebs 500 mcg	0.144	Sando K	0.077
Calcichew D3	0.077	Isosorbide mononitrate 10mg	0.038	Sando phosphate	0.164
Calcichew/calcium carbonate	0.093	Isosorbide mononitrate 20mg	0.032	Senna 7.5mg	0.058
CalcichewD3 Forte	0.071	Isosorbide mononitrate 40mg	0.043	Seretide evohaler 125	0.292

Calcipotriol Ointment	0.193	Isosorbide mononitrate MR 60mg	0.375	Seretide accuhaler 100	0.300
Calcium and ergocalciferol	0.545	Ivabradine 7.5mg	0.717	Seretide accuhaler 250	0.583
Calogen	0.021	K CEE L syrup	0.015	Seretide accuhaler 500	0.682
Candesartan 16mg	0.051	Lacri-lube ointment	0.840	Seretide evohaler 250	0.496
Candesartan 2mg	0.163	Lactulose solution	0.005	Sertraline 50mg	0.056
Carbamazepine M.R.200mg	0.093	Lansoprazole 15mg	0.037	Simple linctus	0.004
Carbocisteine 375mg	0.108	Lansoprazole 30mg	0.046	Simvastatin 20mg	0.030
Carbocisteine liquid 250/5ml	0.028	Latanoprost eye drops 50mcg/ml	0.700	Simvastatin 40mg	0.034
Celluvisc 1% eye drops	0.100	Laxido	0.142	Slow K 600mg	0.043
Cetirizine 10mg	0.032	Lercanidipine 10mg	0.053	Sodium chloride flush 5 mls	0.211
Chlorhexidine Gluconate 0.2% mouthwash	0.012	Lercanidipine 20mg	0.058	Sodium valproate MR 500mg	0.202
Chlorpheniramine 4 mg	0.030	Levofloxacin 500mg	1.832	Solifenacin 5mg	0.921
Ciprofibrate 100mg	3.954	Levothyroxine 100mcg	0.066	Spironalactone 100mg	0.081
Ciprofloxacin 500mg	0.099	Levothyroxine 25mcg	0.096	Sulphasalazine suspension 250mg/5ml	0.085
Ciprofloxacin 750mg	0.800	Levothyroxine 50mcg	0.066	Symbicort 200/6	0.317
Citalopram 10mg	0.030	Lidocaine 5% patch	2.413	Symbicort 400/12	0.633
Citalopram 20mg	0.033	Lisinopril 2.5mg	0.029	Tamiflu 75mg	1.541
Citalopram 40mg	0.037	Lisinopril 20mg	0.034	Tamsulosin MR 400mcg	0.349
Clarithromycin 500mg	0.194	Lisinopril 5mg	0.030	Tazocin iv 4.5g	12.90
Clarithromycin 500mg iv	9.450	Loperamide oral solution 1mg/5ml	0.012	Teicoplanin 200 iv	0.140
Clarithromycin suspension 250mg/5ml	0.068	Loratidine 10mg	0.032	Temazepam 10mg	0.209
Clenil Modulite 100mcg	0.037	Lorazepam 1mg	0.084	Temazepam Elixir 10mg/5ml	0.354
Clopidogrel 300mg	0.233	Losartan 100mg	0.042	Terbinafine 250mg	0.102
Clopidogrel 75mg	0.058	Losartan 25mg	0.034	Terbutaline turbohaler	0.069
Clotrimazole 1% cream	0.058	Losartan 50mg	0.035	Theophylline M/R 300mg	0.085
Co-amoxiclav suspen sugar free 250mg/5ml	0.750	Magnesium Aspartate sachets 243mg	0.895	Theophylline M/R 400mg	0.101
Co-amoxiclav 625mg	0.136	Magnesium Sulphate 2 mmol/ml	5.834	Theophylline M/R 200mg	0.053
Co-amoxiclav iv 1.2g	1.060	Meropenem 1g iv	15.35	Thiamine 100mg	0.116
Co-amilofruse 2.5/20mg	0.107	Mesalazine SR 1g	0.615	Ticagrelor 90mg	0.975
Co-amilofruse 5/40mg	0.105	Metformin 1g MR	0.152	Tigecycline iv 50mg	32.31
Co-careldopa 25mg/100mg	0.157	Metformin 500mg	0.045	Tinzaparin 23,000u (40,000u vial)	34.20
Co-codamol 8mg/500mg	0.034	Metoclopromide 10mg	0.030	Tinzaparin 10,000u	5.950
Codeine 15mg	0.037	Metoclopromide iv 10mg	0.323	Tinzaparin 11,000u (12000u syringe)	7.140

Codeine 30	0.043	Metoprolol 50mg	0.063	Tinzaparin 12,000u	7.140
Codeine linctus 15mg/5ml	0.009	Metronidazole 400mg	0.065	Tinzaparin 18,000u	10.71
Co-dydramol 30mg/500mg	0.122	Metronidazole 500mg iv	3.100	Tinzaparin 2,500u	1.980
Colecalciferol 20,000u	0.967	Miconazole oral gel	0.055	Tinzaparin 3,500u	2.771
Colecalciferol 800 u (Fultium)	0.120	Micralax enema	0.406	Tinzaparin 4,500u	3.563
Colomycin neb	5.600	Midazolam s/c 5mg/5mls	0.600	Tinzaparin 8,000u	4.760
Colpermin capsules	0.122	Mirtazapine 15mg	0.050	Tiotropium (respimat) 2.5mcg	0.542
Combivent nebs	0.397	Mirtazapine 30mg	0.049	Tiotropium 18mcg	1.117
Co-trimoxazole 960mg	0.235	Mirtazapine 45mg	0.062	Tirofiban 250 mcg/ml iv	146.1
Creon 10,000	0.129	Mometasone nasal spray	0.017	Tizanidine 4mg	0.233
Creon 40,000	0.418	Monomil XL 60mg	0.375	Tolterodine 2mg	0.045
Cyclizine iv	1.730	Montelukast 10mg	0.063	Tramadol 50mg	0.033
Cyclizine oral	0.093	Morphine iv 10mg	0.936	Trimethoprim 100mg	0.033
Dexamethasone 2mg	0.982	Morphine Sulphate 30mg	0.208	Trimethoprim 200mg	0.215
Diazepam 2mg	0.031	Morphine sulphate M.R. 10mg	0.087	Trospium 20mg	0.317
Diclofenac Gel 1.16%	0.056	Morphine sulphate M.R. 60mg	0.405	Ultibro Breezhaler	1.083
Difflam mouthwash	0.022	Moxonidine 200mcg	0.061	Uniphyllin MR 200mg	0.053
Dihydrocodeine 30mg	0.048	Mupirocin ointment	1.297	Uniphyllin MR 400mg	0.101
Diltiazem M/R 180mg	0.237	Naseptin nasal cream	0.149	Varenciline 1mg	0.975
Diltiazem M/R 240mg	0.411	Nefopam	0.406	Venlafaxin 75mg	0.047
Diltiazem MR 120mg	0.185	Nicorandil 10mg	0.048	Verapamil 40mg	0.024
Diltiazem MR 90mg	0.130	Nicorandil 20mg	0.097	Viscotears	0.260
Docusate sodium 100 mg	0.070	Nicotine inhalater 15mg	0.756	Vitamin B Co	0.062
Dosulepin 75mg	0.056	Nicotine Gum 4mg	0.107	Warfarin 1mg	0.030
Doxycycline 100mg	0.121	Nicotine patch 14mg	1.343	Warfarin 3mg	0.032
Duoresp 160/4.5	0.250	Nicotine patch 21mg	1.424	Warfarin 5mg	0.033
Duoresp 320/9	0.500	Nicotine patch 7mg	1.303	Water for injections 2ml	0.275
Enalapril 20mg	0.056	Nicotine patch 25mg	1.481	Zomorph 10mg	0.058
Enalapril 10mg	0.039	Nifedipine MR 40mg	0.480	Zomorph 30mg	0.138
Ensure	0.009	Nifedipine SR 30mg	0.245	Zomorph 60 mg	0.270
Ensure compact	2.988	Nitrofurantoin 50mg	0.334	Zopiclone 3.75mg	0.050
Ensure plus juice	0.009	Nystatin suspension 100,000 units/ml	0.079	Zopiclone 7.5mg	0.048
Ensure plus milk	0.006	Octenison wash	0.005		
Epilim Chrono MR 300mg	0.175	Olanzapine 10mg	0.094		

Accident and Emergency attendances

These were costed from the NHS Reference Costs (2015) (Department of Health 2015).[1] An alert was sent to the research team when a patient attended accident and emergency. To ensure no episodes were missed, patients were asked to keep a record of their attendances (in case they attended accidence and emergency in a different healthcare trust) and all electronic records were reviewed after the follow up period.

Table E2. Accident and emergency attendances

Type of attendance	Cost (£)*	Source
A+E attendance VB01Z	377.9	NHS reference cost 2015. EM type 1. Service code VB01Z.
A+E attendance VB02Z	347.5	NHS reference cost 2015. EM type 1. Service code VB02Z.
A+E attendance VB03Z	252.3	NHS reference cost 2015. EM type 1. Service code VB03Z.
A+E attendance VB04Z	227.0	NHS reference cost 2015. EM type 1. Service code VB04Z.
A+E attendance VB05Z	189.5	NHS reference cost 2015. EM type 1. Service code VB05Z.
A+E attendance VB06Z	133.7	NHS reference cost 2015. EM type 1. Service code VB06Z.
A+E attendance VB07Z	164.1	NHS reference cost 2015. EM type 1. Service code VB07Z.
A+E attendance VB08Z	153.0	NHS reference cost 2015. EM type 1. Service code VB08Z.
A+E attendance VB09Z	108.4	NHS reference cost 2015. EM type 1. Service code VB09Z.
A+E attendance VB10Z	112.5	NHS reference cost 2015. EM type 1. Service code VB10Z.
A+E attendance VB11Z	90.2	NHS reference cost 2015. EM type 1. Service code VB11Z.

*Costs inflated for 2015-16 prices

Primary and secondary care outpatient attendances

These were costed from the NHS Reference Costs (2015) (Department of Health 2015).[1] Patients kept a record of outpatient attendances (including attendances at external healthcare trusts), and this was cross-referenced with electronic medical records. If a patient cancelled a clinic attendance, no cost was allocated. If a patient did not attend their appointment without informing clinic, the cost was allocated.

Table E3. Cost of attendance

Type of attendance	Cost (£)*	Source
Anaesthetic clinic, consultant	109.4	NHS reference cost 2015. Service code 190 consultant.
Anaesthetic clinic, nurse	87.9	NHS reference cost 2015. Service code 190 non consultant.
Cardiology clinic, consultant	142.4	NHS reference cost 2015. Service code 320 consultant.
Clinical psychology	195.4	NHS reference cost 2015. Service code 656 non consultant.
Dietetics clinic	71.2	NHS reference cost 2015. Service code 654 non consultant.
Endocrine clinic, consultant	158.2	NHS reference cost 2015. Service code 302 consultant.
ENT clinic, nurse	72.7	NHS reference cost 2015. Service code 120 non consultant.
Gastroenterology clinic, consultant	141.7	NHS reference cost 2015. Service code 301 consultant.
General surgery clinic, consultant	140.5	NHS reference cost 2015. Service code 100 consultant.
GP attendance	44.6	Curtis 2015, 11.7m patient contact.
GP attendance, bloods by nurse	11.2	Curtis 2015, 15.5m patient contact.
GP attendance, practice nurse review	11.2	Curtis 2015, 15.5m patient contact.
Haematology clinic, consultant	164.2	NHS reference cost 2015. Service code 303 consultant.
Haematology ward attendance	347.5	NHS reference cost 2015. Service code DCRDN, band 2.
Maxillo-Facial surgery clinic, consultant	115.4	NHS reference cost 2015. Service code 144 consultant.
Neurosurgery clinic, consultant	215.4	NHS reference cost 2015. Service code 150 consultant.
Oncology clinic, consultant	173.1	NHS reference cost 2015. Service code 370 consultant.
Ophthalmology clinic, technician	64.8	NHS reference cost 2015. Service code 130 non consultant.
Ophthalmology clinic, consultant	97.4	NHS reference cost 2015. Service code 130 consultant.
Orthopaedic clinic, consultant	116.3	NHS reference cost 2015. Service code 110 consultant.
Orthopaedic clinic, nurse	93.7	NHS reference cost 2015. Service code 110 non consultant.
Physiotherapy rehabilitation session	39.2	NHS reference cost 2015. Service code 342 non consultant.
Plastic surgery clinic, consultant	95.6	NHS reference cost 2015. Service code 160 consultant.
Podiatry clinic, podiatrist	39.7	NHS reference cost 2015. Service code 653 non consultant.
Respiratory clinic, consultant	165.9	NHS reference cost 2015. Service code 340 consultant.
Respiratory clinic, nurse	120.4	NHS reference cost 2015. Service code 340 non consultant.
Respiratory clinic, nurse	120.4	NHS reference cost 2015. Service code 340 non consultant.
Respiratory clinic, oxygen nurse	120.4	NHS reference cost 2015. Service code 340 non consultant.
Urology clinic, consultant	103.5	NHS reference cost 2015. Service code 101 consultant.
Urology clinic, nurse	78.0	NHS reference cost 2015. Service code 101 non consultant.

*Costs inflated for 2015-16 prices

Diagnostic tests

These were costed from the NHS Reference Costs (2015) (Department of Health 2015). [1] Tests were recorded from medical electronic records (covering primary and secondary care), and cross referenced with records maintained by patients.

Table E4. Cost of diagnostic tests

Type of test	Cost (£)*	Source
24 hour tape	155.0	NHS reference cost 2015. Currency code EY51Z
CT one area, post contrast	173.3	NHS reference cost 2015. Currency code RD21A
CT scan pelvis	114.5	NHS reference cost 2015. Currency code RD23Z 2 areas without contrast
CT two areas, with contrast	156.0	NHS reference cost 2015. Currency code RD24Z 2 areas with contrast
DEXA bone scan	59.8	NHS reference cost 2015. Currency code RD50Z
Diagnostic endoscopic upper GI tract	510.7	NHS reference cost 2015. Currency code FZ60Z
ECG	18.4	Galasko GI, Barnes SC, Collinson P, et al. What is the most cost-effective strategy to screen for left ventricular systolic dysfunction: natriuretic peptides, the electrocardiogram, hand-held echocardiography, traditional echocardiography, or their combination? European heart journal. 2006 Jan;27(2):193-200.
Echocardiogram	65.9	NHS reference cost 2015. Currency code RD51A
Lower endoscopy	373.9	NHS reference cost 2015. Currency code FZ51Z
MRCP liver	138.8	NHS reference cost 2015. Currency code RD01A outpatient
MRI one area, no contrast	127.7	NHS reference cost 2015. Currency code RD01A MRI one area no contrast
MRI one area, post contrast only	431.6	NHS reference cost 2015. Currency code RD02A MRI one area post contrast
Plain film x-ray	30.4	NHS reference cost 2015. Currency code DAPF.
Sleep study	212.8	NHS reference cost 2015. Currency code DZ50Z
Spirometry	54.7	52 for 2012 secondary care from "HTA report 2015" (for multiplier look in PRSSU). Adjusted 2014/15
Tilt room test	142.9	Krahn AD, Klein GJ, Yee R, et al. Cost implications of testing strategy in patients with syncope: randomized assessment of syncope trial. J Am Coll Cardiol. 2003 Aug 6;42(3):495-501. PubMed PMID: 12906979.
Ultrasound	64.8	NHS reference cost 2015. Currency code RD40Z less than 20 minutes

*Costs inflated for 2015-16 prices

Laboratory tests

Individual laboratory tests and add-on costs were provided by the trust.

Table E5. Cost of laboratory tests

Laboratory test name	Cost	Laboratory test name	Cost	Laboratory test name	Cost
ABG (arterial blood gas)	6.56	GGT	0.53	PTH	7.86
Albumin	0.47	HbA1c	1.49	Random urine protein	1.33
Alpha-1-Antitrysin	8.79	HEP B surface	9.12	Retics	3.90
Amylase	0.85	HIB Antibody	16.39	Rheumatoid factor	1.86
Antigens (urine)	42.7	Immunoglobulins	4.65	Routine faeces	19.99
Blood bank	7.23	INR	4.53	Serum immunofixation	31.12
Blood cultures	14.7	Intrinsic factor	10.41	Sputum culture	7.59
Blood film	3.39	Lactate	1.13	T3	2.44
Blood glucose	0.51	LFTs	2.44	T4	2.11
blood ketones	6.31	Lipids	0.41	TB culture	27.74
Bone profile	1.48	Magnesium	0.54	Theophylline	23.28
Calcium	0.52	Mixing studies APTT	4.59	Total CK	1.01
Cholesterol	0.51	MRSA rejected sample	5.93	TRF saturation	1.23
Coagulation screening	7.46	MRSA screen	7.66	Troponin T (HS)	4.73
CRP	0.73	Mycoplasma	10.62	TSH	1.95
D-Dimer	19.06	NT pro BNP	19.33	Unsuitable sample	6.00
EGFR	0.47	Osmolality	3.84	Urate	0.80
ESR	3.86	Paraprotein screen	3.44	Urea and electrolytes	2.58
FBC	3.90	Phosphate	0.51	Urine immunofixation	31.12
Ferritin	4.51	Point of care FBC	21.46	Urine Microscopy and culture	6.58
Flu assay (PCR)	57.92	Procalcitonin	17.62	Vitamin B12	4.82
Folate	4.55	Prolactin	3.49	Vitamin D	9.86
Gastric parietal cell antibody	9.75	Prostate specific antigen	2.82		

Health care staff

The Personal Social Services Research Unit's *Unit Costs of Health and Social Care 2015* cost compendium was used to allocated costs.[2] All contacts included add on costs and qualifications.

Table E6. Cost of contact with healthcare staff

Type of healthcare worker	Cost (£)*	Source
Community psychiatric nurse	20.1	PSSRU Unit costs of health and social care 2015
Dietician band 5	39.2	PSSRU Unit costs of health and social care 2015
Dietician band 6	44.5	PSSRU Unit costs of health and social care 2015
Dietician home visit	11.0	PSSRU Unit costs of health and social care 2015, 15m
District nurse home visit	12.5	PSSRU Unit costs of health and social care 2015, 15m
Doctor consultant	153.0	PSSRU Unit costs of health and social care 2015
Doctor F1	42.5	PSSRU Unit costs of health and social care 2015
Doctor F2	50.1	PSSRU Unit costs of health and social care 2015
Doctor ST1	64.7	PSSRU Unit costs of health and social care 2015
Doctor ST2	67.0	PSSRU Unit costs of health and social care 2015
Doctor registrar ST3	68.4	PSSRU Unit costs of health and social care 2015
Doctor registrar ST4	70.0	PSSRU Unit costs of health and social care 2015
Doctor registrar ST5	71.7	PSSRU Unit costs of health and social care 2015
Doctor registrar ST6	73.3	PSSRU Unit costs of health and social care 2015
Doctor registrar ST7	75.0	PSSRU Unit costs of health and social care 2015
GP doctor home visit	43.4	PSSRU Unit costs of health and social care 2015
GP doctor phone call	27.1	PSSRU Unit costs of health and social care 2015
GP nurse home visit	11.1	PSSRU Unit costs of health and social care 2015
NHS 111	8.0	Department of health, NHS 111
Occupational therapist	39.2	PSSRU Unit costs of health and social care 2015
Occupational therapist home visit	22.1	PSSRU Unit costs of health and social care 2015, 30m
Pharmacist, band 6	50.2	PSSRU Unit costs of health and social care 2015
Physio home visit, band 6	11.1	PSSRU Unit costs of health and social care 2015
Physiotherapist band 5	39.2	PSSRU Unit costs of health and social care 2015
Physiotherapist band 6	44.5	PSSRU Unit costs of health and social care 2015
Physiotherapist band 7	49.5	PSSRU Unit costs of health and social care 2015
Psychology	52.2	PSSRU Unit costs of health and social care 2015
Rapid response team	51.2	PSSRU Unit costs of health and social care 2015
Respiratory Specialist Nurse Band 6	46.2	PSSRU Unit costs of health and social care 2015
Respiratory Specialist Nurse Band 7	60.2	PSSRU Unit costs of health and social care 2015
Respiratory Specialist Nurse Band 8a	68.6	PSSRU Unit costs of health and social care 2015
Social worker	57.2	PSSRU Unit costs of health and social care 2015
Speech and language therapist, band 5	38.1	PSSRU Unit costs of health and social care 2015
Support worker, band 2	24.1	PSSRU Unit costs of health and social care 2015

We recorded start and end times for 105 medical clerkings for patients within this RCT, which averaged 0.75 hours for a non-consultant and 0.33 for a consultant. These averages were used in instances where no end time was stated. Inpatient reviews were assumed to be 15 minutes in duration, unless otherwise documented, again based on average review times. The duration of reviews during the Hospital at Home period was recorded for all visits, which included travel time and time spent with the patient.

The PSSRU unit of costs does not provide the hourly rate for registrars of different grade, but provides information on calculating costs.[2] The actual salaries for doctors of all grades was calculated by obtaining basic salaries, adding a 50% banding supplement and adjusting for a 48 hour week. The salary on-costs were changed accordingly based on the actual salary.

For the Respiratory Specialist Nurses the average cost of the band 6, 7 and 8a nurses was calculated (several new appointments were made over the time course of the study, so most band 6 nurses were at the low end of the band 6 pay scale, whilst band 7 and 8a nurses were at the top end). The salary on-costs were changed accordingly based on the actual salary. The hourly rate for inpatient reviews and home visits by RSNs was the same.

Inpatient ward stay costs

The cost of a day in hospital was costed by the trust. This included direct, indirect and overhead costs. Certain costs were removed to ensure they were not double counted, such as drug costs and pharmacy input.

For the cost of a day on the medical admissions unit, an average day was obtained from costs over a two year period (2013-2015) and adjusted for current prices. In the instance that a patient spent less than a day on the unit, an hourly rate was applied. The cost of a rehab bed day was calculated in the same way.

The cost of a day on a respiratory ward was calculated from two difference respiratory wards over a 2 year period (2013-2015) to give an average bed day cost.

Table E7. Ward cost

Ward stay type of cost per bed day	Cost (£)	Source
Medical admissions unit	294.9	Healthcare trust
Medical ward	246.2	Healthcare trust
Rehabilitation ward	168.8	Healthcare trust

Other inpatient stays/ procedures

Some patients had inhospital stays at other healthcare trusts, and so the cost of the episode was obtained from the provider and/ or a tariff cost was used.

Table E8. Cost of other inpatient stays/ procedures

Reason for admission	Cost (£)	Source
Intermediate hip procedure for trauma	7452.3	NHS reference cost 2015. Currency code HT14A.
Osteonecrosis	2383.0	Cost of admission provided by external healthcare trust
Myocardial infarction with percutaneous intervention	4613.0	Cost of admission provided by external healthcare trust
Myocardial infarction with percutaneous intervention, short stay	2554.0	Cost of admission provided by external healthcare trust
Urinary retention	1881.0	Cost of admission provided by external healthcare trust
Crohn's disease	2763.5	NHS reference cost 2015. Currency code FZ37K X1.0132
Minor hip procedure for trauma	2517.5	NHS reference cost 2015. HT15Z. X1.0132
Surgical tooth removal	462.0	Cost of admission provided by external healthcare trust

Oxygen therapy

Patients who received oxygen therapy either already had long term oxygen therapy in place or were given a hospital oxygen concentrator to avoid delays in returning home. The cost of LTOT therapy is £2.29 per day, adjusted to current prices.[3] The cost of the hospital oxygen concentrator is £0.57 per day (Airsep Visionaire = £900, plus five-year warranty and maintenance = £140). The Respiratory Specialist Nurse delivered and set up the oxygen and provided education, which was captured separately within the patient visit time. We had concerns that using the cost £0.57 was too conservative, therefore we used a daily cost of oxygen at £2.29 per day for everyone. The additional cost of running a home oxygen assessment and review services were captured elsewhere.

Non-invasive ventilation

The cost of non-invasive ventilation was calculated based on the cost of a machine over a five year period, and the costs of other equipment. NIV is provided on a respiratory support unit with higher staffing costs, and 1.5 hours of nursing time was included. Other care/ treatment over and above the average patient with ECOPD in terms of physiotherapy, doctors, diagnostic tests, laboratory tests (including arterial blood gas analysis) and medication was captured separately.

Table E9. Cost of non-invasive ventilation

Type of cost	Unit cost (£)	Cost per day (£)
V60 Ventilator (machine, servicing, etc.)	12000	6.6
Circuit (tubing and mask changed weekly)	50.8	7.3
Filter (changed x2 per week)	2.7	0.8
Nebuliser T piece	1.3	0.2
Face shield (used one in three episodes)	77.7	3.7
cleaning of machine- band 6, 10 mins, once per episode	8.5	2.4
Nurse time (1.5 hours extra per patient per day) band 6	51	76.5
Overall (includes adjustment to 2015/16)		98.7

Table E10. Utility scores in complete case analysis at baseline, 14 days and 90 days, and total QALYs.

	HAH	UC
Baseline utility mean (SD)	0.520 (0.275)	0.499 (0.245)
14 day utility mean (SD)	0.606 (0.274)	0.557 (0.329)
90 day utility mean (SD)	0.523 (0.282)	0.507 (0.346)
Total QALYs, unadjusted (SD)	0.139 (0.059)	0.132 (0.069)
Total QALYs, adjusted for baseline utility (SD)	0.138 (0.052)	0.133 (0.052)
Total QALYs, MI, unadjusted (SD)	0.138 (0.058)	0.129 (0.072)
Total QALYs, MI, adjusted for baseline utility (SD)	0.137 (0.053)	0.130 (0.053)

Table E11. Difference between Hospital at Home and usual care health status with complete case analysis and multiple imputation

	Difference between HAH and UC (complete case)	Difference between HAH and UC (multiple imputation)
HADS-A 14 day	-1.5	-2
HADS-A 90 day	0	0
HADS-D 14 day	-1	-1
HADS-D 90 day	-0.5	-0.5
CAT 14 day	-1	-0.5
CAT 90 day	-2	-1.5
Utility 14 day (EQ-5D-5L)	0.036	0.051
Utility 90 day (EQ-5D-5L)	-0.002	-0.014

*Values are median differences, except for utility which is mean. Improvements in health status are negative for HADS and CAT, and positive for utility scores. The Minimal Clinically Important Difference is 1.5 for HADS-A and HADS-D, 2 for CAT and 0.051 for the EQ-5D-5L.

Table E12. Costs associated with the index admission

	HAH, £	UC, £
Bed stay	385.3	1040.1
Inpatient healthcare review	186.8	266.1
Diagnostic tests	69.4	101.3
Laboratory tests	67.4	70.0
NIV costs	9.87	73.2

Table E13. Numbers of inpatient interactions with healthcare workers

	HAH	UC
Inpatient interactions, n	1158	1558
Consultant, n	292	271
Doctor (not consultant), n	408	543
Physiotherapy, n	221	484
Resp Specialist Nurse, n	85	58
Occupational therapy, n	14	43
Dietician, n	12	6

Figure E1. Cost-effectiveness plane (A) and cost-effectiveness acceptability curve (B), multiple imputation.

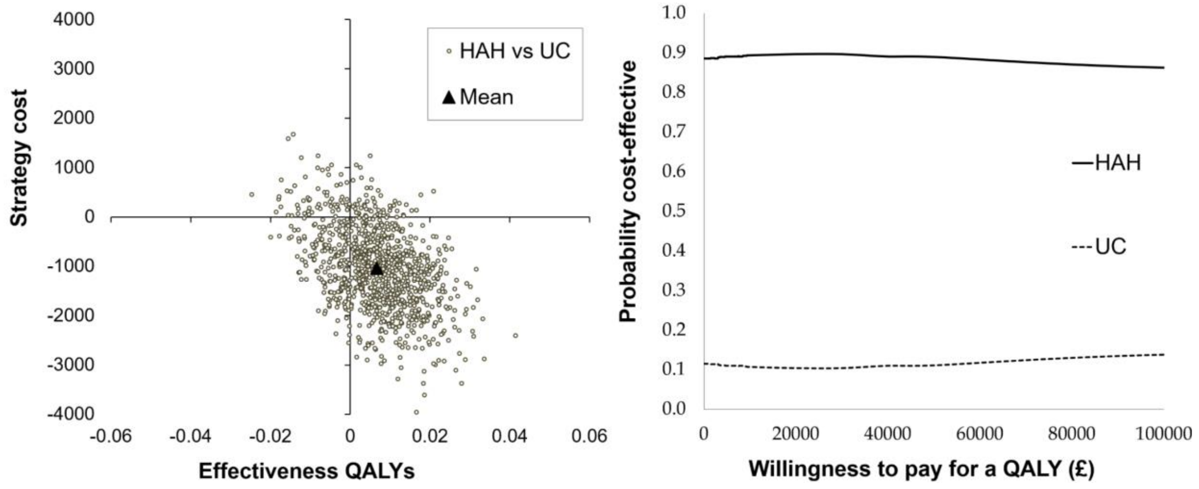

Table E14. Characteristics of decliners, and patients in HAH and UC

	Decliners	HAH	UC
Female	54.3	53.3	51.7
Age (SD)	70.6 (9.8)	71.0 (9.6)	68.7 (10.5)
Pack years (IQR)	40 (40-60)	45 (35-50)	44 (30-60)
FEV1 % pred (SD)	45.1 (19.6)	45.5 (18.4)	42.1 (16.3)
DECAF 1, %	65.7	71.7	53.4
eMRCD 5a, %	8.6	20	15.5
CXR consolidation, %	25.7	25	15.5
pH less than 7.35, %	2.9	10	10.3
PaO2 kPa (SD)	8.0 (7.35 to 9.0)	7.6 (7.2-9.3)	7.9 (7.2-10.2)
One previous admission 12 months, %	22.9	20	20.7
Two previous admissions 12 months, %	25.7	35	32.8
Prior social care, %	5.7	5	1.7
IHD, %	25.7	23.3	20.7
Diabetes, %	17.1	13.3	8.6
LV dys, %	5.7	1.7	5.2
Anxiety, %	11.4	15	5.2
Depression, %	14.3	20	15.5

Online supplement references

E1. Department of Health. NHS reference costs collection guidance for 2015 to 2016. In: Health Do, editor. UK: Department of Health; 2015.

E2. Curtis L. Unit Costs and Social Care. Personal Social Services Research Unit. 2015.

E3. Loveman E, Copley VR, Colquitt J, et al. The clinical effectiveness and cost-effectiveness of treatments for idiopathic pulmonary fibrosis: a systematic review and economic evaluation. Health technology assessment (Winchester, England). 2015 Mar;19(20):i-xxiv, 1-336. PubMed PMID: 25760991. Pubmed Central PMCID: PMC4781002. Epub 2015/03/12. eng.
